# A Simple and Robust Statistical Method to Define Genetic Relatedness of Samples Related to Outbreaks at the Genomic Scale – Application to Retrospective *Salmonella* Foodborne Outbreak Investigations

**DOI:** 10.3389/fmicb.2019.02413

**Published:** 2019-10-24

**Authors:** Nicolas Radomski, Sabrina Cadel-Six, Emeline Cherchame, Arnaud Felten, Pauline Barbet, Federica Palma, Ludovic Mallet, Simon Le Hello, François-Xavier Weill, Laurent Guillier, Michel-Yves Mistou

**Affiliations:** ^1^ANSES, Laboratory for Food Safety, Université PARIS-EST, Maisons-Alfort, France; ^2^Unité des Bactéries Pathogènes Entériques, Institut Pasteur, Centre National de Référence des Salmonella, Paris, France

**Keywords:** outbreak investigation, *Salmonella* Typhimurium, monophasic *S*. Typhimurium (*S*. 1, 4, [5], 12:i:-), cgMLST, wgMLST, SNPs, genes, kmers

## Abstract

The investigation of foodborne outbreaks (FBOs) from genomic data typically relies on inspecting the relatedness of samples through a phylogenomic tree computed on either SNPs, genes, kmers, or alleles (i.e., cgMLST and wgMLST). The phylogenomic reconstruction is often time-consuming, computation-intensive and depends on hidden assumptions, pipelines implementation and their parameterization. In the context of FBO investigations, robust links between isolates are required in a timely manner to trigger appropriate management actions. Here, we propose a non-parametric statistical method to assert the relatedness of samples (i.e., outbreak cases) or whether to reject them (i.e., non-outbreak cases). With typical computation running within minutes on a desktop computer, we benchmarked the ability of three non-parametric statistical tests (i.e., Wilcoxon rank-sum, Kolmogorov–Smirnov and Kruskal–Wallis) on six different genomic features (i.e., SNPs, SNPs excluding recombination events, genes, kmers, cgMLST alleles, and wgMLST alleles) to discriminate outbreak cases (i.e., positive control: C+) from non-outbreak cases (i.e., negative control: C−). We leveraged four well-characterized and retrospectively investigated FBOs of *Salmonella* Typhimurium and its monophasic variant *S*. 1,4,[5],12:i:- from France, setting positive and negative controls in all the assays. We show that the approaches relying on pairwise SNP differences distinguished all four considered outbreaks in contrast to the other tested genomic features (i.e., genes, kmers, cgMLST alleles, and wgMLST alleles). The freely available non-parametric method written in R has been designed to be independent of both the phylogenomic reconstruction and the detection methods of genomic features (i.e., SNPs, genes, kmers, or alleles), making it widely and easily usable to anybody working on genomic data from suspected samples.

## Introduction

New genome sequencing technologies provide an unparalleled, powerful tool for the characterization of infectious agents. In the field of food safety, genomic analyses have taken an essential place in the investigation of foodborne outbreaks (FBOs) (Mole, 2013). The many studies focusing on retrospective analyses of well-characterized FBOs have firmly established that phylogenetic reconstruction based on whole genome sequencing (WGS) allows for the investigation of epidemic clusters with a previously unmatched resolution (Nadon et al., 2017). The advantages of WGS have been tested for the main bacterial foodborne pathogens: *Salmonella* (den Bakker et al., 2014), *Listeria* (Hilliard et al., 2018), *E. coli* (Holmes et al., 2015), and *Campylobacter* (Rokney et al., 2018). In all cases, WGS-based approaches proved to be more accurate and discriminating than traditional typing methods like pulsed-field gel electrophoresis (PFGE) or multi-locus VNTR analysis (MLVA). Through WGS-based subtyping, cases were correctly identified and additional clinical isolates, not considered at the time of the initial investigation that was performed with traditional typing methods, can even be identified.

Several genomic investigations into FBOs have shown that the level of genetic diversity within a FBO depends on the history of the contamination and its investigation (Stimson et al., 2019). Many studies have concluded that the concept of a general threshold of single nucleotide polymorphism (SNP) is not operational even within the same serovar (Pightling et al., 2018). The nature of the outbreak (i.e., sources, dissemination, and duration) affects the genetic distances between isolates and requires a more subtle definition of outbreak cases. The history of the isolates (i.e., origin, matrix, sampling date, and context) must be carefully examined because the evolution rate can vary in different food matrices or food-processing environments (Duchêne et al., 2016). Epidemiological data and traceback information are essential to rebuild the epidemic events (Pightling et al., 2018; Sanaa et al., 2019), however, they may contain inaccurate and missing data about the history of isolates. In addition, significant evolutionary events can obscure the relatedness of isolates (Snitkin et al., 2011). In spite of these caveats, the isolates belonging to the same recent FBO are genetically similar, and phylogenomic methods are suitable to trace the source, dissemination routes and mode of contamination. From this perspective, the generation of a phylogenomic tree is a commonly used method (den Bakker et al., 2010; Holmes et al., 2015; Hilliard et al., 2018; Rokney et al., 2018). The traditional phylogenetic reconstructions based on orthologous genes and multi-gene alignments are today increasingly replaced by inferences based on pairwise distances. It is possible to compute matrices of pairwise distances from a diversity of genomic features: SNPs (Timme et al., 2017), genes (Page et al., 2015), kmers (Ondov et al., 2016), or alleles from coregenome and whole genome multi-locus sequencing typing (i.e., cgMLST and wgMLST) (Chen et al., 2017).

A crucial aspect of all molecular typing investigations resides in the capacity to build a relevant and strong outgroup: a set of isolates genetically close yet not directly related to the sanitary situation of interest. A way to proceed is to include in the analysis a large number of isolates sampled in the same period and geographical area of the epidemic isolates and, when the information is available, belonging to the same or a close genetic group. In fact, at present, the construction of this control group is largely empirical and built on common sense principles.

Here, we propose a non-parametric statistical approach to distinguish between outbreak and non-outbreak cases as an alternative to methods based on pairwise differences thresholds, bootstrap estimations or visual inspections of phylogenetic trees (Lee et al., 2015a,b). We extracted six genomic features at the coregenome, accessory genome or pangenome scales from genomic data of four historical *Salmonella enterica* outbreaks, and we evaluated the ability of three non-parametric tests—Wilcoxon rank-sum (WS), Kolmogorov–Smirnov (KS), and Kruskal–Wallis (KW)—to discriminate between outbreak and non-outbreak cases.

## Materials and Methods

### Selection of Outbreaks and Isolates for Retrospective Epidemiological Investigations

**FIGURE 1:**
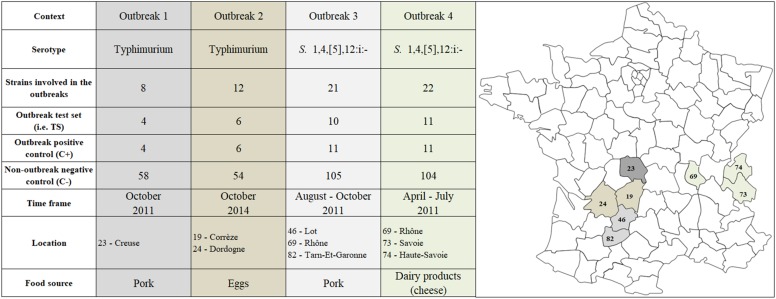
Localization in France and description of strains belonging to *S.* Typhimurium (*n* = 66) *S*. 1,4,[5],12:i:- (*n* = 126) involved in the four foodborne outbreaks of interest. These strains were selected from the National Reference Center for *Salmonella* (NRC), ANSES *Salmonella* Network and “Direction générale de l’alimentation” as part of the French Ministry of Agriculture, Food and Forestry.

Four *Salmonella* FBOs with complete epidemiological information and available microbiological materials were selected for the study (Supplementary Table S1). Two outbreaks (#1 and #2) were due to *S.* Typhimurium and the two others (#3 and #4) to *S.* 1,4,[5],12:i:- (Figure 1). The outbreaks occurred in France between 2010 and 2014, and isolates were obtained from patients, contaminated food, animals and the environment (Figure 1). The strain collection corresponding to the four outbreaks included 63 strains (Supplementary Table S1) to which we added 129 non-outbreak controls presenting the same PFGE pattern for most of them collected through passive surveillance (Supplementary Data S1 and Supplementary Table S1). The clinical strains were obtained from the National Reference Center (NRC) for *Salmonella* at the “Institut Pasteur Paris.” Food, animal and environmental strains were obtained from the ANSES *Salmonella* Network at the French Food Safety Laboratory in Maisons-Alfort. The antigenic formulae were determined by glass slide agglutination according to the White-Kauffmann-Le Minor scheme (Grimont and Weill, 2007), and PCRs were performed following EFSA recommendations to confirm that all *S.* 1,4,[5],12:i:- isolates were monophasic variants of serovar Typhimurium (EFSA, 2010; Tennant et al., 2010). The sequence types (ST) were predicted using the version 2.16.1 of the program mlst developed by Seemann T^1^. based on components of the PubMLST website^2^, integrating BIGSdb developed by Jolley and Maiden (Jolley and Maiden, 2010).

### Genomic DNA Preparation and Sequencing

Genomic DNA was prepared from 2 ml of BHI overnight cultures with the Wizard^®^ Genomic DNA Purification Kit (Promega, France), according to the manufacturer’s instructions for gram-negative organisms. Gels of 0.8% agarose were used to assess the genomic DNA integrity. The DNA concentration was measured with a Qubit^®^ fluorimeter and the purity ratio was assessed with a Nanodrop^®^ Spectrophotometer. Library preparation and NGS sequencing were performed by the “Institut du Cerveau et de la Moelle épinière” (ICM^3^, Hôpital de la Pitié-Salpêtrière, Paris). The libraries were prepared with NextEra XT technology (Illumina), indexed according to the manufacturer recommendations (Illumina), purified with the Agencourt AMPure XP system (Beckman Coulter) and quantified with the Microfluidic Labchip GX (PerkinElmer). The sequencing was performed with 300 cycles High Output kit v2 cartridges (i.e., 800 million of paired-end reads of 150 bases) and a NextSeq 500 sequencer.

### Genomic Analysis

With an objective to evaluate which genetic information performs the best in a context of outbreak investigations with non-parametric approaches, we used a series of genomic features of pairwise differences at the coregenome (i.e., SNPs including or excluding recombination events and cgMLST), accessory genome (i.e., presence-absence of genes) and pangenome (i.e., kmers and wgMLST) scales.

#### Variant Calling (SNPs and InDels)

The coregenome SNPs and small InDels were detected based on the variant caller HaplotypeCaller that was implemented in the iVARCall2 workflow (Felten et al., 2017), used *Salmonella* Typhimurium LT2 (NCBI NC_003197.1) as a reference genome and followed the best practices proposed by the Genome Analysis ToolKit (GATK) (McKenna et al., 2010). More precisely, secondary alignments around small InDels were performed and duplications were excluded before variant calling analysis via local *de novo* assembly of haplotypes in active regions. The matrices of pairwise SNP differences and pseudogenomes were computed using in-house Python scripts called ‘VCFtoMATRIX’ and ‘VCFtoPseudoGenome,’ respectively. The pseudogenomes correspond to the reference genome where the genotypes of detected variants were replaced in each genome (Felten et al., 2017). As previously described, variants from homologous recombination events (>400 bp) were detected with ClonalFrameML (Didelot and Wilson, 2015) and subsequently excluded, or kept, with the script ‘Clonal_VCFilter’ (Felten et al., 2017).

#### Allelic Differences at the Coregenome Scale (cgMLST)

Allelic differences were computed with BioNumerics v.7.6.3 software (Applied Maths, Sint-Martens-Latem, Belgium) using a combination of assembly-free and assembly-based allele calling. A similarity threshold of ≥85% was used for assembly-based calls and gapped alignments were allowed. The cgMLST *Salmonella* scheme integrated within the software consists of a total of 3 002 *loci*. The cgMLST was restricted to ≥80% homology in ≥95% of the isolates (Vincent et al., 2018). The matrices of pairwise allele differences were obtained with a scaling factor of 1 and a limit of differences ≤200. The alleles displaying discrepancy between the assembly-free and assembly-based analyses were excluded. Finally, the allelic differences were computed on 2 620 and 2 723 *loci* for *S.* Typhimurium and its monophasic variant *S.* 1,4,[5],12:i:-, respectively.

#### Gene Differences at the Accessory Genome Scale

The assembly was performed with an in-house workflow called ARTwork, based on coverage control (i.e., >100X) with Bbmap (Bushnell, 2014), read normalization (i.e., 100X) with Bbnorm (Xu et al., 2015), quality control of reads with FastQC (Andrews, 2010), read trimming (i.e., >20 of Quality Control) with Trimmomatic (Bolger et al., 2014), *de novo* assembly with SPAdes (Bankevich et al., 2012), selection of closely related genome with MinHash (Ondov et al., 2016), scaffolding with MeDuSa (Bosi et al., 2015), gap filling with GMcloser (Kosugi et al., 2015), trimming of small scaffolds (i.e., <200 bases) with Biopython (Cock et al., 2009) as well as control of assembly quality with QUAST (Gurevich et al., 2013) and MultiQC (Ewels et al., 2016). Based on these draft genomes, pangenomes of both genome datasets were constructed with Roary (Page et al., 2015) setting 95% of identity for blastp and a strict definition of the coregenome (i.e., 100% of isolates with coregenes); the paralogs were kept for downstream analyses. The matrices of pairwise gene differences were produced with an in-house Python script called ‘roary_to_pairwise.’

#### kmer Differences

Using the genome assemblies obtained as described above, an in-house Python workflow called QuickPhylo was developed in order to produce matrices of pairwise kmer differences based on a form of locality-sensitive hashing called MinHash (Indyk and Motwani, 1998) implemented in Mash (Ondov et al., 2016). More precisely, Mash is run for each genome against a sketch including all the studied genomes, and the shared hashes produced are retained to create matrices of pairwise kmer differences, setting Mash with 1 000 selected kmers of 15 bases in order to perform a fast (i.e., 1 000 kmers in the sketch) and discriminant computing (i.e., smallest bounded error with kmers of 15 bases according to simulated data representative of the genome size of *S. enterica*), respectively. It must be noted that the single-copy kmers were included, assuming those kmers are not artifacts.

#### Allelic Difference at the Pangenome Scale (wgMLST)

Allelic differences were computed according to the wgMLST scheme with the BioNumerics v.7.6.3 software (Applied Maths, Sint-Martens-Latem, Belgium) as explained above. The wgMLST *Salmonella* scheme integrated within the software consists of a total of 15 874 loci. The matrices of pairwise allele differences were computed on 3 530 and 3 698 *loci* for *S.* Typhimurium and its monophasic variant *S.* 1,4,[5],12:i:-, respectively.

### Statistical Approaches

**FIGURE 2:**
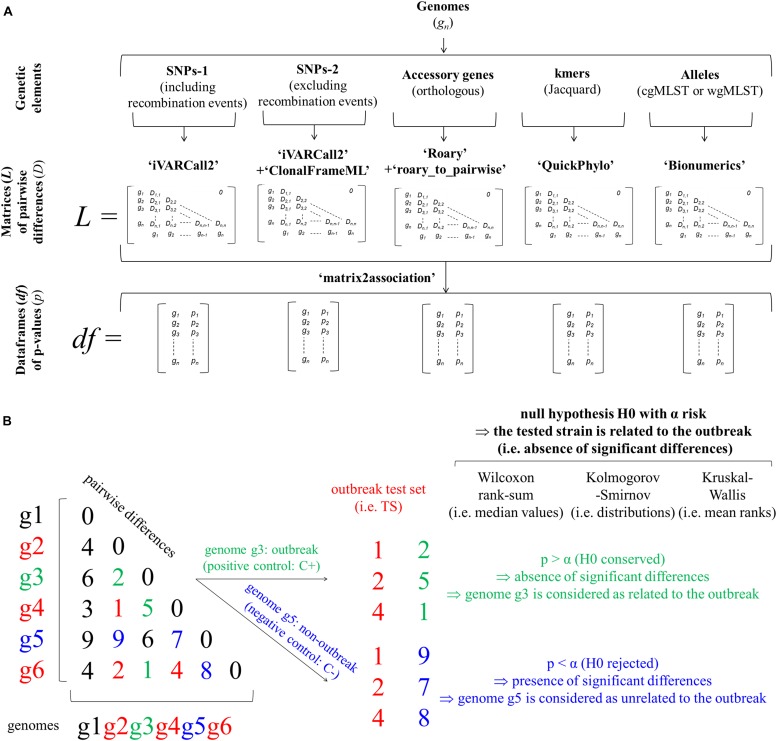
Workflows implementing non-parametric tests to assess statistical differences of pairwise differences of genomic features at the core (i.e., approaches ‘SNPs-1,’ ‘SNPs-2,’ and ‘cgMLST’), accessory (i.e., approaches ‘genes’) and pangenome (i.e., approaches ‘kmers’ and ‘wgMLST’) scales in order to investigate food poisoning outbreaks **(A)** and statistical approaches implementing non-parametric tests (i.e., WS, KS, and KW) comparing pairwise differences of genomic features during a foodborne outbreak (FBO) investigation **(B)**. The approaches ‘SNPs-1,’ ‘SNPs-2,’ ‘genes,’ ‘kmers,’ ‘cgMLST,’ and ‘wgMLST’ were performed with the workflows iVARCall2 with or without ClonalFrameML, ARTWork-Roary, ARTWork-QuickPhylo, and BioNumerics (Applied Maths), respectively. The ‘SNPs’ approaches were performed including (i.e., ‘SNPs-1’) or excluding (i.e., ‘SNPs-2’) SNPs from recombination events identified with ClonalFrameML. The R script ‘matrix2association’ estimates statistical differences between two lists of pairwise differences existing across genomes known to be involved in a studied outbreak (i.e., outbreak test set: TS) and between these genomes and a tested genome (i.e., outbreak control C+ or non-outbreak control C–) in order to assign (i.e., absence of statistical differences: H0 conserved), or not (i.e., presence of statistical differences: H0 rejected) this tested genome to the outbreak of interest.

The statistical approach includes three successive steps (Figure 2A). Based on input genomes (i.e., g_n_ in Figure 2A), the first step of the statistical approach aims to identify the genetic features of interest. More precisely, the identification of coregenome SNPs, including (i.e., SNP-1) or excluding (i.e., SNP-2) recombination events detected with ClonalFrameML, accessory genes (i.e., orthologous genes), kmers (i.e., 1 000 selected kmers) and alleles (cgMLST or wgMLST) are performed with the workflows detailed above: iVARCall 2, ARTWork-Roary, ARTWork-QuickPhylo and BioNumerics (Applied Maths), respectively (Figure 2A). Based on these workflows, the second step corresponds to the production of matrices (i.e., *L* in Figure 2A) of pairwise differences (i.e., *D* in Figure 2A) regarding the considered genomic features (i.e., SNPs-1, SNPs-2, accessory genes, kmers, cgMLST, or wgMLST alleles). The third step is a computation step that divides each pairwise difference matrix of interest into two lists of pairwise differences, which are then compared by three non-parametric tests based on the R script ‘matrix2association’ (i.e., *p*_n_ in Figure 2A). The first list corresponds to pairwise differences existing across genomes known to be involved in the outbreak. The second list corresponds to pairwise differences existing between these outbreak genomes and the tested genome. With the hypothesis that the tested genome is related to the outbreak of interest (i.e., null hypothesis H0: absence of significant differences), this script estimates statistical differences between these two lists of pairwise differences. Both lists are compared with the three non-parametric tests in order to assign (i.e., H0 conserved), or not (i.e., H0 rejected), the tested genome to the outbreak of interest (Figure 2B). The non-parametric two-sample WS, KS, and KW [i.e., R Stats package (R Development Core Team, 2015)] tests assess the statistical differences of median values, distributions and mean ranks, respectively (Figure 2B). These non-parametric tests were selected because the distributions and equality of variances were not known. In practice, two groups of outbreak (i.e., positive control: C+) and non-outbreak (i.e., negative control: C−) controls were formed for each outbreak and tested in a pairwise manner using these non-parametric tests against a third group representative of samples involved in the studied FBOs (i.e., outbreak test set: TS). The two groups of samples involved in the studied FBOs (TS) and outbreak control (C+) were previously confirmed to be epidemiologically involved in the outbreaks of interest (Figures 1, 2B). Following the results of statistical tests, the C+ and C− were assigned (i.e., H0 conserved and tested sample considered as related) or not (i.e., H0 rejected and tested sample considered as unrelated) to the outbreak of interest (TS). In addition, the developed R script ‘matrix2association’ produced graphical representations of the distributions of pairwise differences. The dataframes of *p*-values (i.e., *df* in Figure 2A) were plotted with ggplot2 and used to choose the most suitable method(s) [i.e., genomic features(s) combined with non-parametric test(s)] (Wickham, 2009).

### Phylogenomic Inference

Phylogenomic inferences were performed by maximum likelihood based on pseudogenomes produced by the iVARCall2 workflow and the general time-reversible (GTR) model implemented in the RaxML program (Stamatakis, 2014). In addition to the nucleotide substitution model (GTR) and the secondary structure 16-state model, models describing rate variation among sites were also applied. Gamma distribution (G) and convergences of the phylogenomic inferences were checked based on rapid bootstrap analysis (Stamatakis et al., 2008). The phylogenomic inferences and annotations were graphically represented with ggtree R package (Yu et al., 2017).

## Results

### Assessment of Genomic Data Quality

The results of non-parametric approaches are supported by the good quality of the mapping and assembly of pseudo- and draft- genomes (Supplementary Data S3A and Supplementary Table S2). The presence of exogenous DNA was assessed based on the cumulated size of scaffolds, GC content, genome fraction, gene content as well as the logarithmic and hyperbolic forms of the curves representing the new and conserved genes according to the sizes of genome datasets, respectively (Supplementary Data S3 and Supplementary Table S2). One sample of *S.* Typhimurium 10CEB498SAL appeared as potentially contaminated (i.e., total length of 6.26 Mb) and was deliberately not excluded from the study in order to demonstrate that the non-parametric approaches applied on pairwise differences of genomic features provide robust results independently of all the other tested genomes. In summary, both genome datasets of *S.* Typhimurium and *S.* 1,4,[5],12:i:- presented similar pangenome constitutions (Table 1).

**TABLE 1:** Pangenome constitutions of genome datasets belonging to *S.* Typhimurium (*n* = 66) and *S*. 1,4,[5],12:i:- (*n* = 126) involved in the four foodborne outbreaks of interest.

**Localization of genes**	**Range of genomes (%)**	**Number of genes**	
		***S.* typhimurium**	***S.* 1,4,[5],12:i:-**
Core	[100,100]	3 794	4 066
Soft core	[95,100[	501	387
Shell	[15,95[	520	209
Cloud	[0,15[	3 192	2 006
Total	[0,100[	8 007	6 668

*Assembly and pangenome analyses were performed with ARTWork and Roary, respectively. Paralogs were retained for downstream analyses.*

### Evaluation of the Considered Genomic Features to Distinguish Outbreak (i.e., Positive Control: C+ and Non-outbreak (i.e., Negative Control: C−) Controls

**FIGURE 3:**
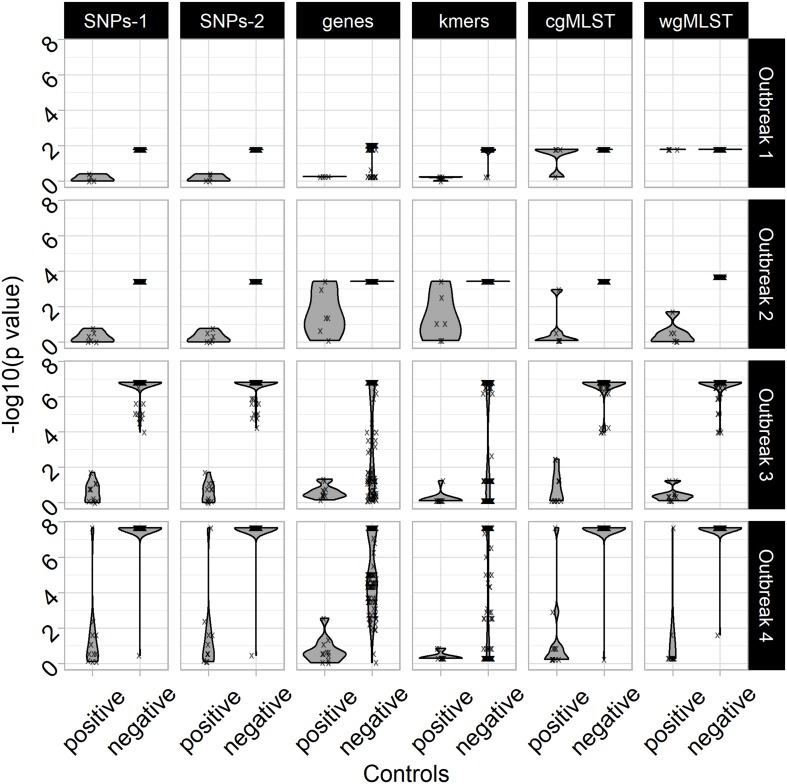
Negative common logarithms of *p*-values from non-parametric tests: Kolmogorov–Smirnov assessing statistical differences of pairwise differences of genomic features at the core (i.e., approaches ‘SNPs’ and ‘cgMLST’), accessory (i.e., approaches ‘genes’) and pangenome (i.e., approaches ‘kmers’ and ‘wgMLST’) scales in order to investigate food poisoning outbreaks of 192 *S.* Typhimurium (i.e., outbreaks #1 and #2; *n* = 66) and *S*. 1,4,[5],12:i:- (i.e., outbreaks #3 and #4; *n* = 126). The ‘SNPs’ approaches were performed including (i.e., ‘SNPs-1’) or excluding (i.e., ‘SNPs-2’) SNPs from recombination events identified with ClonalFrameML. The R script ‘matrix2association’ estimates statistical differences between two lists of pairwise differences existing across all genomes known to be involved in a studied outbreak (i.e., outbreak test set: TS) and between these genomes and a tested genome (i.e., outbreak control C+ or non-outbreak control C–) in order to assign (i.e., absence of statistical differences: H0 conserved), or not (i.e., presence of statistical differences: H0 rejected), this tested genome to the outbreak of interest. The approaches ‘SNPs-1,’ ‘SNPs-2,’ ‘genes,’ ‘kmers,’ ‘cgMLST,’ and ‘wgMLST’ were performed with the workflows iVARCall2 with and without ClonalFrameML, ARTWork-Roary, ARTWork-QuickPhylo, and BioNumerics (Applied Maths), respectively.

To assess the value of the non-parametric statistical approaches for FBO analysis, we built four datasets corresponding to four FBOs that took place in France between 2010 and 2014 (Figure 1 and Supplementary Data S2). For each FBO, our approach consisted of building two groups of C+ and C− isolated in the same period of time and comparing them to a set of strains involved in FBOs of interest (TS) (Figure 1). For each genomic feature (i.e., ‘SNPs-1,’ ‘SNP-2,’ ‘genes,’ ‘kmers,’ ‘cgMLST,’ and ‘wgMLST’), matrices of pairwise differences were obtained including all isolates, and the R script ‘matrix2association’ was run to extract lists of pairwise distances and evaluate the genetic relatedness (Figure 2) existing between genomes from TS and these genomes against each tested genome from the C+ and C− (Figure 3).

In the present study, all the *p*-values from non-parametric tests define the likelihood of incorrectly rejecting the null hypothesis: the absence of statistical differences between samples from TS and the C+ or C− genomes (i.e., [TS against TS] versus [TS against C+] or [TS against TS] versus [TS against C−]). In other words, the statistical tests estimate the probability of excluding a sample that actually belongs to the outbreak. The important result is that, for all four outbreaks, the use of non-parametric tests on pairwise SNP differences (i.e., genomic features SNP-1 and SNP-2) provides clear discrimination between C+ and C− samples (Figure 3). The use of the genomic feature ‘SNPs-1’ allows for the distinguishing of C+ from C− regardless of the non-parametric test used (Supplementary Data S4 and Supplementary Tables S3, S4). This approach allows a straightforward grouping of C+, while all C− stay apart.

Interestingly, the SNP-based non-parametric approaches (SNP-1 or SNP-2) allow for the distinguishing between C+ and C− even when the TS contained only four isolates (outbreak #1) (Figures 1, 3). Additionally, the range of *p*-values of the SNP-based non-parametric approaches, including (i.e., SNP-1) or excluding the recombination events (i.e., SNP-2), indicated that the discrimination between C+ and C− genomes was improved when more genomes were included in the TS of outbreak #1 (i.e., 4), #2 (i.e., 6), #3 (i.e., 10), and #4 (i.e., 11) (Figure 3 and Supplementary Data S4). By contrast, the use of pairwise differences of ‘genes,’ ‘kmers,’ or ‘wgMLST’ resulted in overlapping ranges of *p*-values between C+ and C−, meaning a higher alpha risk of incorrectly rejecting the null hypothesis (Supplementary Data S4 and Supplementary Tables S3, S4). It is interesting to note that the genomic feature ‘cgMLST’ was found to be as efficient as SNP-1’ and ‘SNP-2’ for the outbreaks #2, #3, and #4 (Figure 3 and Supplementary Data S4). This result suggests that the genomic feature ‘cgMLST,’ combined with the non-parametric tests, is accurate when at least six genomes are present in the TS (Figures 1, 3).

An interesting feature arose from the analysis of outbreak #4, where the sample 2013LSAL03045 was associated with C+ (i.e., range of *p*-values: 2.8 × 10^–2^ to 3.4 × 10^–1^) while it was initially positioned in C−. The sample 2013LSAL03045 was isolated from the environment (i.e., water off-take) 2 years after outbreak #4 (i.e., July 2011 versus 17 July 2013) in a different geographical area (i.e., Rhône Alpes versus Normandie) (Supplementary Table S3). Although no epidemiological evidence relates it to the outbreak, the statistical analysis brings it closer to the epidemic samples, suggesting that the SNP-based non-parametric approaches can reveal unexpected links. This approach is also able to detect erroneous epidemiological assignation. For instance, the sample 11CEB5591SAL was rejected from C+ in outbreak #4 (i.e., *p*-values between 2.1 × 10^–8^ and 1.6 × 10^–7^). This sample corresponded to a soil sample isolated in the same period and from the same region that was mistakenly linked to strains responsible for infections, and it was included in the C+ in our study (Supplementary Table S3).

### Considerations on Non-parametric Tests

By considering the SNP-based non-parametric approach, we found that the range of *p*-values defining the C+ and C− were similar between the WS, KS, and KW tests. For instance the *p*-values defining the C+ in outbreak #3 ranged between 2.3 × 10^–2^ and 9.8 × 10^–1^, 1.9 × 10^–2^ and 9.9 × 10^–1^ as well as 3.9 × 10^–2^ and 8.4 × 10^–1^, while those referring to C− ranged between 4.5 × 10^–7^ and 6.4 × 10^–5^, 1.6 × 10^–7^ and 1.0 × 10^–4^ as well as 8.4 × 10^–7^ and 1.2 × 10^–4^ for WS, KS, and KW tests, respectively (Supplementary Data S4 and Supplementary Tables S3, S4). With the notable exceptions of samples 2013LSAL03045 (i.e., expected C− and identified as C+) and 11CEB5591SAL (i.e., expected C+ and identified as C−) mentioned above, all the other tested genomes (i.e., 345) were successfully assigned as C+ and C− with the SNP-based non-parametric approaches (i.e., ‘SNPs-1’) implementing WS, KS or KW tests (Supplementary Data S4 and Supplementary Tables S3, S4).

### Effect of the Recombination Events on the SNP-Based Non-parametric Approaches

Homologous recombination events may increase the number of SNPs in the impacted genomic regions. This phenomenon may thus shift the distributions of pairwise SNP differences and hinder the non-parametric comparisons of pairwise SNP differences. In order to assess the impact of homologous recombination events, we performed the SNP-based non-parametric approaches including (i.e., ‘SNP-1’) or excluding (i.e., ‘SNP-2’) the recombination events. Overall, 13 and four recombination events were detected with ClonalFrameML (Didelot and Wilson, 2015) across genomes of *S.* Typhimurium (i.e., ranging from 404 to 1 194 bp) and its monophasic variant (i.e., ranging from 532 to 14 597 bp), respectively. Starting with SNP datasets of 4 818 for *S.* Typhimurium and 3 204 for its monophasic variant, the exclusion of recombination events led to datasets of 4 797 and 3 154 SNPs. The SNP-based non-parametric approaches provide an accurate assignment of all tested genomes (i.e., 345) to C+ and C− with either SNPs-1 or SNPs-2 genomic features (Supplementary Data S4). In summary, the method was not impacted by the presence of recombination events in our dataset of genomes.

### Reproducibility of the SNP-Based Non-parametric Approaches

The non-parametric comparisons of pairwise SNP differences existing between the genomes may be impacted by the selection of genomes in the outbreak test sets (TS). We tested this hypothesis by running the SNP-based non-parametric approach for each outbreak with three additional randomized replicates of TS. For all repeated trials, all isolates (i.e., 345) were accurately assigned to outbreak (C+) or non-outbreak (C−) controls (Supplementary Data S5). From this random resampling, we can conclude that the TS composition does not affect the predictive power of the SNP-based non-parametric approaches, and the method is consequently robust.

### Phylogenomic Reconstruction and Non-parametric Approaches

**FIGURE 4:**
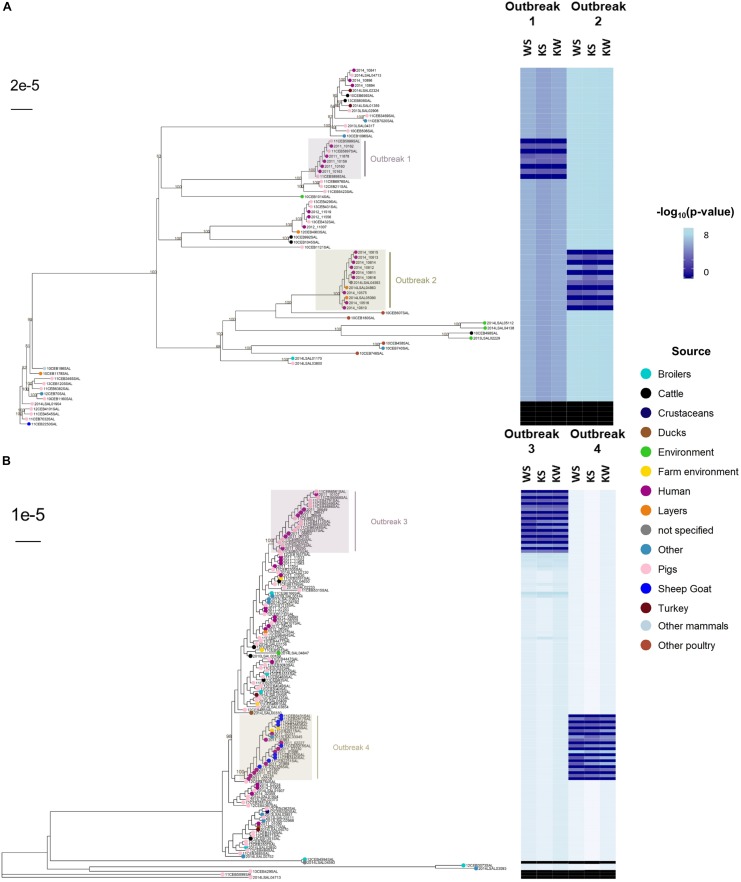
Phylogenetic inference based on coregenome single nucleotide polymorphisms (SNPs) identified in 192 *Salmonella enterica* subsp. *enterica* during outbreaks in France caused by serovars Typhimurium (**A**: outbreaks #1 and #2; *n* = 66) and *S*. 1,4,[5],12:i:- (**B**: outbreaks #3 and #4; *n* = 126) and related *p*-values from non-parametric tests WS (i.e., differences of median values), KS (i.e., differences in distributions) and KW (i.e., differences of mean ranks) aiming to access statistical differences of pairwise SNP differences (i.e., approaches ‘SNPs-1’ including recombination events). The R script ‘matrix2association’ estimates statistical differences between two lists of pairwise differences existing across all genomes known to be involved in a studied outbreak (i.e., outbreak test set: TS) and between these genomes and a tested genome (i.e., outbreak control C+ or non-outbreak control C–) in order to assign (i.e., absence of statistical differences: H0 conserved), or not (i.e., presence of statistical differences: H0 rejected), this tested genome to the outbreak of interest. The SNPs were identified by the workflow ‘iVARCall2’ against the reference genome *S.* Typhimurium LT2 (NCBI NC_003197.1). The produced pseudogenomes (4,857,432 bp) were inferred using the program ‘RaxML’ based on a bootstrap analysis and search for best-scoring Maximum Likelihood tree with General Time-Reversible model of substitution and the secondary structure 16-state model. Bootstraps higher than 80% are represented at each node.

The reconstruction of a phylogenomic tree is one of the most frequently used methods to combine genomic information and extract evidence during outbreak investigations. To support the idea that the non-parametric approach reflects and can easily replace phylogenetic inference to establish the genetic relatedness of isolates, we performed SNP-based phylogenetic reconstruction, including recombination events, and used the tree to report the *p*-values computed with the WS, KS, or KW tests (Figure 4). The results depict epidemiological clades perfectly delineated from context and control samples.

## Discussion

### Practical Aspects for the Use of Non-parametric Tests

With regards to our exhaustive comparison of approaches (‘SNPs-1,’ ‘SNPs-2,’ ‘genes,’ ‘kmers,’ ‘cgMLST,’ and ‘wgMLST’), we recommend the application of the WS, KS, or KW tests on pairwise SNP differences in order to distinguish between outbreak (C+) and non-outbreak (C−) genomes against genomes from confirmed cases (i.e., outbreak test set: TS). In the context of real-time outbreak investigations, and according to Nadon et al. (2017), the genomes unrelated to the FBO of interest (C−) have to be chosen based on epidemiological information (i.e., time and place of isolation as close as possible to that of the outbreak) or genomic proximity using a rapid method like the kmer approach. From our results, the number of genomes in the outbreak test set (TS) seem to influence the contrast between C+ and C− *p*-values. Consequently, the more samples in the TS the better the discrimination will be. In a real situation, the number of samples available to investigators will define the TS. However, it is important to highlight that even with a small number of samples in TS (i.e., 4 in outbreak #1) the performance of the non-parametric approach was fully satisfactory.

Some bottlenecks caused by the non-parametric approach in terms of computational and time requirements remain the same as with phylogenomic methods: (i) the generation of high quality genome assembly to obtain ‘genes,’ ‘kmers,’ ‘cgMLST,’ and ‘wgMLST’ data and (ii) the various calling steps to extract ‘SNPs-1’ and ‘SNP-2’ data (Figure 2). However, the non-parametric approach makes it possible to eliminate one of the longest and most complex steps: phylogenetic reconstruction. While running the R script ‘matrix2association,’ the non-parametric test outcome is almost instantaneous. For instance, our Linux network is constituted of 43TB for storage and has 240 threads distributed across five servers for computing power. This Linux network allows the execution of assembly (i.e., ARTWork) and variant calling (iVARCall2) for 96 *Salmonella* genomes in around 400 min. Both assembly and variant calling represent a similar duration of execution.

Our results also show that the non-parametric tests cannot confidently distinguish between outbreak C+ and C− controls when the ‘genes,’ ‘kmers,’ or ‘wgMLST’ genomic features were used, while the ‘cgMLST’ genomic feature combined with the non-parametric tests allowed for the accurate assignation of controls when TS contained at least six genomes (i.e., outbreak#2, #3, and #4). By contrast, the SNP genomic features joined to these non-parametric tests were successful even when TS contains only four genomes (outbreak#1), as was also supported by several trials of randomly selected genomes in TS (Supplementary Data S5). This lower discriminatory power of the cgMLST compared to the SNP genomic feature might be due to the fact that the SNP genomic feature includes intergenic and intragenic core variants (Felten et al., 2017), whereas the cgMLST only integrates core alleles defined from coding sequences (Pearce et al., 2018).

To date, our conclusions are supported by the datasets of samples tested in this study. Other datasets will have to be analyzed in order to generalize the method we adopted in this study. It is therefore important that genomic outbreak reference datasets grow in volume and diversity (Timme et al., 2017). Publicly available reference datasets can be used for method validation and to gain knowledge of pathogen evolution over the course of outbreaks. Consequently, our method is a complementary process through which to compile and verify these datasets.

### Impact of the Rate of Nucleotide Evolution

All DNA-based phylogenetic tree reconstructions use explicit statistical models of nucleotide evolution (Yang and Rannala, 2012). The molecular clock does not always tick regularly and variation in substitution rates may occur for subpopulations of pathogens experiencing different environmental conditions (Okoro et al., 2012; Hawkey et al., 2013; Mather et al., 2013). It is currently not clear if variations occurring during FBO are due to drift (i.e., neutral evolution) or to a selection process. Moreover, no outbreak is like any other. The period during which a pathogen linked to a given source circulates is highly variable, ranging from a few days (Taylor et al., 2015) to several years (Lee et al., 2015a). The proposed non-parametric method theoretically solves these issues (i.e., evolution rate and/or outbreak duration) because it estimates the statistical differences of pairwise differences existing between genomes from the outbreak test set [TS against TS] and pairwise differences existing between these genomes and a tested genome (i.e., [TS against C+] or [TS against C−]). That is, these parameters are sampled, represented and considered within the dataset. If both lists of pairwise differences increase proportionally because of the evolutionary rate or FBO duration (i.e., [TS against TS] and [TS against C+]), the non-parametric test would be able to correctly conserve (i.e., absence of differences: [TS against TS] versus [TS against C+]) or reject (i.e., presence of differences [TS against TS] versus [TS against C−]) the null hypothesis. On the other hand, the environments encountered may be conducive to growth or, on the contrary, may limit it, and this information will in most cases be missing during the investigation. These uncertainties and the heterogeneity of these situations are likely to affect genome evolution. These elements led to the conclusion that the definition of threshold values, below which isolates would be epidemiologically linked, is not of good practice, at least regarding FBO investigations. Remaining attentive to the epidemiological traceback information is of major importance before assuming connections between isolates of different origins (Pightling et al., 2018), hence their recommendation to be careful about bootstrap support and tree topology in the context of phylogenetic approaches. For these reasons, we proposed a non-parametric approach independent of pairwise difference thresholds; however, considering the questionable assignment of samples 11CEB5591SAL and 2013LSAL03045, we support the conclusion that a good FBO investigation requires sound epidemiological information.

### Dealing With Recombination Events in the Outbreak Test Set

The impact of recombination events occurring during an outbreak can artificially increase the pairwise differences between related samples. This is an important technical issue in genomic investigations. Thus, other authors studying the largest outbreak of *Legionella pneumophila* in Germany strongly recommended compensating for recombination to distinguish related and unrelated genomes of the same sequence type based on cgMLST (Petzold et al., 2017). Similarly, the National Institutes of Health (NIH) in the United States demonstrated that genomes of *Acinetobacter baumannii* strains involved in nosocomial infections belonged to the same epidemic lineage, though they have diverged into three sub-lineages mainly driven by homologous recombination events across 20% of their genomes (Snitkin et al., 2011). This recommendation also applies to the non-parametric approach; if recombination events only appear in a C+ genome the likelihood of wrong assignment to C− would increase, and the method could fail to assign this sample to the TS. Although the *Salmonella* datasets and statistical approaches used in the present study are relatively insensitive to recombination events, we recommend that recombination events from the SNP dataset are excluded to avoid theoretically spurious assignments.

### Independency to the Phylogenomic Inferences

One of the main difficulties in the more widespread use of genomics is the variety of procedures and bioinformatics workflows used to reconstruct sequences and establish genetic relatedness between strains. Food and environmental reference laboratories are facing requests from health services to link clinical strains to food and environmental strains originating from epidemiological inquiries or surveillance networks. The objective of any molecular investigation of FBO is to establish links based on genetic relatedness between clinical and food isolates while distinguishing them from the circulating unrelated population. In these situations, the availability of general guidance to assess the genetic relatedness between isolates would be of great help. Few current studies compare fast and inaccurate phylogenomic clustering methods based on distances (e.g., Neighbour-joining, Unweighted pair group method with arithmetic mean) to slow and accurate phylogenomic clustering methods based on characters (e.g., maximum likelihood and Bayesian) (Lees et al., 2018). Faced with the contemporary debate about biological veracities and technical feasibility of distances- versus character-based methods during real-time investigations of FBO (Sneath and Sokal, 1973), our non-parametric approach presents the crucial advantage of being completely independent of the phylogenomic reconstruction methods. Many different approaches are implemented for genomic analysis of pathogens in the context of public health investigations. There is still no evidence that this complex situation will simplify in the near future. Rather, it is likely that a variety of approaches—‘SNPs’ (i.e., the most accurate), ‘genes’ (i.e., the most *de novo*), ‘alleles’ (i.e., the most portable) and ‘kmers’ (i.e., the fastest)— implemented in a variety of pipelines will coexist. A large number of benchmarking studies that evaluate methods testify to this complex situation. Thus, to continue the implementation of WGS approaches in the field of food safety, there is a need for methods that allow for a reliable quantification of the genetic relatedness between strains and which maintain dialogue between laboratories using different pipelines.

### What’s Next?

Although transmission dynamics of several outbreaks were successfully solved thanks to high-resolution genomics, the contemporary challenge is to describe ongoing outbreaks in real time based on genomic epidemiology and to lead safety authorities and public health decision makers to consider the implementation of automated and integrated genomic systems (Tang and Gardy, 2014). Many initiatives are moving in this direction. The Pathogen detection browser of the GenomeTrakr international genomic reference database of foodborne pathogens from food and environmental isolates provides a cluster analysis on a daily basis (Timme et al., 2019), as it is also the case for PulseNet International network dedicated to laboratory-based surveillance for food-borne pathogens (Nadon et al., 2017). In Europe, genomic surveillance of gastrointestinal infections is implemented on a routine basis by Public Health England (Mook et al., 2018) or the Austrian Agency for Health and Food Safety (Pietzka et al., 2019). Transmission events may be described by rooted phylogenomic reconstructions with ancient branches presenting clusters of genomes associated with specific hosts or environmental compartments. However, rooted phylogenomic reconstructions during an ongoing outbreak cannot be considered as directional transmission trees because of the poor statistical support of nodes closed to the final leaves. A recent algorithm based on a reversible jump Monte-Carlo Markov Chain proposes a new way to address directional transmission during ongoing transmission (Didelot et al., 2017), and this would consequently improve our proposed non-parametric approaches with a view to provide a fast, discriminant and accurate method that is generally applicable to investigate FBOs.

## Conclusion

The advantages of WGS led food safety laboratories to generate phylogenomic trees and to propose genetic distance thresholds to investigate FBOs. We proposed a novel approach based on non-parametric tests, which is independent of phylogenomic trees reconstruction and thresholds of pairwise distances. The proof of concept was validated by performing a retrospective analysis of four *S.* Typhimurium and S. 1,4,[5],12:i:- FBOs. The approach can be applied to multiple pairwise differences measured at the coregenome (i.e., SNPs or cgMLST), accessory genome (i.e., genes) and pangenome (i.e., kmers or wgMLST) scales.

## Data Availability Statement

The new scripts developed from the present study for genomic and phylogenomic analyses can be found in the following repositories: https://github.com/afelten-Anses/ARtWORK; https://github.com/afelten-Anses/QuickPhylo. The statistical approach developed is available in the https://github.com/lguillier/matrix2association repository. The previously developed script called iVARCall2 (Felten et al., 2017) can be downloaded from the repository https://github.com/afelten-Anses/VARtools/tree/master/iVARCall2 and the corresponding open source package is now available in Conda (https://anaconda.org/itsmeludo/repo and https://conda.io/docs/). Sequencing data is available in the BioProject PRJEB30613 of the European Nucleotide Archive (ENA) (https://www.ebi.ac.uk/ena/data/view/PRJEB30613).

## Author Contributions

NR, SC-S, EC, LG, and M-YM conceived the study and contributed equally to the design and analysis of data. LG, NR, AF, and LM conceptualized algorithms. NR, AF, LM, PB, and EC implemented scripts. NR, EC, and SC-S executed commands. SL and F-XW obtained, selected, and provided clinical strains. NR, LG, FP, SC-S, and M-YM drafted the manuscript. All authors commented and approved the final manuscript, took public responsibility for appropriate portions of the content, and agreed to be accountable for all aspects of the work in terms of accuracy or integrity.

## Conflict of Interest

The authors declare that the research was conducted in the absence of any commercial or financial relationships that could be construed as a potential conflict of interest.

## Footnotes

1 https://github.com/tseemann/mlst

2 https://pubmlst.org/

3 www.icm-institute.org

## Supplementary Material

DATA S1 Epidemiological details about the four foodborne outbreaks of *S.* Typhimurium (**A:** *n* = 66) and *S*. 1,4,[5],12:i:- (**B:** *n* = 126) retrospectively studied. Strains were selected from the collections of the National Reference Center for *Salmonella* (NRC), the ANSES *Salmonella* Network and the “Direction générale de l’alimentation” as part of the French Ministry of Agriculture, Food and Forestry.

DATA S2 Description of the four foodborne outbreaks of interest. Strains were selected from the collections of the National Reference Center for *Salmonella* (NRC), the ANSES *Salmonella* Network and the “Direction générale de l’alimentation” as part of the French Ministry of Agriculture, Food and Forestry.

DATA S3 Number **(A)** and size **(B)** of scaffolds, other parameters of assembly and mapping **(C)** and number of genes resulting from pangenome analyses **(D)** of *Salmonella enterica* subsp. *enterica* (i.e., black bars; *n* = 192) serovars Typhimurium (i.e., gray bars or on the left side; *n* = 66) and *S*. 1,4,[5],12:i:- (i.e., white bars or on the right side; *n* = 126). Assembly, mapping and variant calling, as well as computing of quality metrics and pangenome analyses were performed with ARTWork, iVARCall2, Quast-MultiQC and Roary, respectively. Means and standard deviation are represented.

DATA S4 Likelihoods of non-parametric tests WS (i.e., differences of median values), KS (i.e., differences in distributions) and KW (i.e., differences of mean ranks) assessing statistical differences of pairwise differences of genomic features at the core (i.e., approaches ‘SNPs-1,’ ‘SNP-2,’ and ‘cgMLST’), accessory (i.e., approaches ‘genes’) and pangenome (i.e., approaches ‘kmers’ and ‘wgMLST’) scales in order to investigate food poisoning outbreaks of 192 *S.* Typhimurium (i.e., outbreaks #1 and #2; *n* = 66) and *S*. 1,4,[5],12:i:- (i.e., outbreaks #3 and #4; *n* = 126). The ‘SNPs’ approaches were performed including (i.e., ‘SNPs-1’) or excluding (i.e., ‘SNPs-2’) SNPs from recombination events identified with ClonalFrameML. The R script ‘matrix2association’ estimates statistical differences between two lists of pairwise differences existing across all genomes known to be involved in a studied outbreak (i.e., outbreak test set: TS) and between these genomes and a tested genome (i.e., outbreak control C+ or non-outbreak control C−) in order to assign (i.e., absence of statistical differences: H0 conserved), or not (i.e., presence of statistical differences: H0 rejected), this tested genome to the outbreak of interest. The approaches ‘SNPs-1,’ ‘SNP-2,’ ‘genes,’ ‘kmers,’ ‘cgMLST,’ and ‘wgMLST’ were performed with the workflows iVARCall2 with or without ClonalFrameML, ARTWork-Roary, ARTWork-QuickPhylo, and BioNumerics (Applied Maths), respectively.

DATA S5 Reproducibility of negative common logarithms of *p*-values from non-parametric tests WS (i.e., differences of median values), KS (i.e., differences in distributions) and KW (i.e., differences of mean ranks) assessing the statistical differences of pairwise SNP differences including recombination events (i.e., approach ‘SNP-1’) in order to investigate food poisoning outbreaks of 192 *S.* Typhimurium (i.e., outbreaks #1 and #2; *n* = 66) and *S*. 1,4,[5],12:i:- (i.e., outbreaks #3 and #4; *n* = 126). The R script ‘matrix2association’ estimates statistical differences between two lists of pairwise differences existing across all genomes known to be involved in a studied outbreak (i.e., outbreak test set: TS) and between these genomes and a tested genome (i.e., outbreak control C+ or non-outbreak control C−) in order to assign (i.e., absence of statistical differences: H0 conserved), or not (i.e., presence of statistical differences: H0 rejected), this tested genome to the outbreak of interest. The approach ‘SNPs-1’ was performed with the workflow iVARCall2. In total, four random selections of samples included in the outbreak test set (TS) were performed in order to access reproducibility of the non-parametric approaches.

TABLE S1 Isolates used as outbreak test set (TS) and outbreak control (i.e., positive control: C+), both considered as involved in outbreaks of 63 *S.* Typhimurium (i.e., outbreaks #1 and #2; *n* = 20) and *S*. 1,4,[5],12:i:- (i.e., outbreaks #3 and #4; *n* = 43). The clinical strains were obtained from the National Reference Center (NRC) for *Salmonella* at the “Institut Pasteur Paris.” Food, animal and environmental strains were obtained from the ANSES *Salmonella* Network at the French Food Safety Laboratory in Maisons-Alfort.

TABLE S2 Parameters for the assembly and mapping of the studied genomes of 192 *S.* Typhimurium (i.e., *n* = 66) and *S*. 1,4,[5],12:i:- (i.e., *n* = 126). Assembly, mapping and variant calling as well as computing of quality metrics and pangenome analyses were performed with ARTWork, iVARCall2, Quast-MultiQC, and Roary, respectively. For both assembly and mapping the reference genome was *Salmonella* Typhimurium LT2 (NCBI NC_003197.1).

TABLE S3 Negative common logarithms of p-values from non-parametric tests WS (i.e., differences of median values), KS (i.e., differences in distributions), and KW (i.e., differences of mean ranks) assessing statistical differences of pairwise differences of genomic features at the core (i.e., approaches ‘SNPs-1,’ ‘SNPs-2,’ and ‘cgMLST’), accessory (i.e., approaches ‘genes’) and pangenome (i.e., approaches ‘kmers’ and ‘wgMLST’) scales in order to investigate food poisoning outbreaks of 192 *S.* Typhimurium (i.e., outbreaks #1 and #2; *n* = 66) and *S*. 1,4,[5],12:i:- (i.e., outbreaks #3 and #4; *n* = 126). The ‘SNPs’ approaches were performed including (i.e., ‘SNPs-1’) or excluding (i.e., ‘SNPs-2’) SNPs from recombination events identified with ClonalFrameML. The R script ‘matrix2association’ estimates statistical differences between two lists of pairwise differences existing across all genomes known to be involved in a studied outbreak (i.e., outbreak tested set: TS) and between these genomes and an unknown genome (i.e., outbreak control C+ or non-outbreak control C−) in order to assign (i.e., absence of statistical differences: H0 conserved), or not (i.e., presence of statistical differences: H0 rejected), this tested genome to the outbreak of interest. The approaches ‘SNPs-1,’ ‘SNP-2,’ ‘genes,’ ‘kmers,’ ‘cgMLST,’ and ‘wgMLST’ were performed with the workflows iVARCall2 with and without ClonalFrameML, ARTWork-Roary, ARTWork-QuickPhylo, and BioNumerics (Applied Maths), respectively.

TABLE S4 Mean, standard deviation (i.e., the signs ‘±’), minimum and maximum (i.e., in square brackets) of the negative common logarithms of *p*-values from non-parametric tests WS (i.e., differences of median values), KS (i.e., differences in distributions), and KW (i.e., differences of mean ranks) assessing statistical differences of pairwise differences of genomic features at the core (i.e., approaches ‘SNPs-1,’ ‘SNPs-2,’ and ‘cgMLST’), accessory (i.e., approaches ‘genes’) and pangenome (i.e., approaches ‘kmers’ and ‘wgMLST’) scales in order to investigate food poisoning outbreaks of 192 *S.* Typhimurium (i.e., outbreaks #1 and #2; *n* = 66) and *S*. 1,4,[5],12:i:- (i.e., outbreaks #3 and #4; *n* = 126). The ‘SNP’ approaches were performed including (i.e., ‘SNPs-1’) or excluding (i.e., ‘SNPs-2’) SNPs from recombination events identified with ClonalFrameML. The R script ‘matrix2association’ estimates statistical differences between two lists of pairwise differences existing across all genomes known to be involved in a studied outbreak (i.e., outbreak tested set: TS) and between these genomes and a tested genome (i.e., outbreak control C+ or non-outbreak control C−) in order to assign (i.e., absence of statistical differences: H0 conserved), or not (i.e., presence of statistical differences: H0 rejected), this tested genome to the outbreak of interest. The approaches ‘SNPs-1,’ ‘SNPs-2,’ ‘genes,’ ‘kmers,’ ‘cgMLST,’ and ‘wgMLST’ were performed with the workflows iVARCall2 with and without ClonalFrameML, ARTWork-Roary, ARTWork-QuickPhylo, and BioNumerics (Applied Maths), respectively.
