# Supplementary material for: A Simple and Robust Statistical Method to Define Genetic Relatedness of Samples Related to Outbreaks at the Genomic Scale – Application to Retrospective Salmonella Foodborne Outbreak Investigations
Source: Front Microbiol. 2019 Oct 24;10:2413. doi: 10.3389/fmicb.2019.02413 (PMC6821717; doi:10.3389/fmicb.2019.02413)
Supplement: DATA S1 — Epidemiological details about the four foodborne outbreaks of S. Typhimurium (A: n = 66) and S. 1,4,[5],12:i:- (B: n = 126) retrospectively studied. Strains were selected from the collections of the National Reference Center for Salmonella (NRC), the ANSES Salmonella Network and the “Direction générale de l’alimentation” as part of the French Ministry of Agriculture, Food and Forestry. [file Data_Sheet_1.PDF]

A

Year	**Human**	Outbreaks 1 and 2	**Food**	Pork	Broiler	Other poultry	Turkey	Cattle	Egg	Composite food	**Animal**	Layer	Other poultry	Cattle	Other mammal	**Environment**	Eco-system	**Total**
	**Sample Type**																	
**2010**				3		2		3		2		1	2	1	1		1	16
**2011**		5		9						2								16
**2012**		3										1						4
**2013**				6										1			1	8
**2014**		12		2	1		2		1			2					2	22
**Total**	**20**		**33**								**9**					**4**		**66**

20 human strains
42 non-human strains (animal and food)
4 environmental strains

B

Year	**Human**	Outbreaks 3 and 4	**Food**	Pork	Broiler	Turkey	Duck	Cattle	Other meat not specified	Sheep goat's dairy product	Crustacean	**Animal**	Pigs	Layer	Broiler	Cattle	Sheep Goat	**Environment**	Farm environment	Eco-system	**Total**
	**Sample type**																				
**2009**								1	1												2
**2010**								1											1		2
**2011**		26		23	1		1			7							2		4		64
**2012**		4		15							1		6	1	4	1					32
**2013**				3				1								1				4	9
**2014**		3		6	1	2	1													4	17
**Total**	**33**		**65**									**15**						**13**			**126**

33 human strains
80 non-human strains (animal and food)
13 environmental strains
