# Supplementary material for: A Simple and Robust Statistical Method to Define Genetic Relatedness of Samples Related to Outbreaks at the Genomic Scale – Application to Retrospective Salmonella Foodborne Outbreak Investigations
Source: Front Microbiol. 2019 Oct 24;10:2413. doi: 10.3389/fmicb.2019.02413 (PMC6821717; doi:10.3389/fmicb.2019.02413)
Supplement: DATA S2 — Description of the four foodborne outbreaks of interest. Strains were selected from the collections of the National Reference Center for Salmonella (NRC), the ANSES Salmonella Network and the “Direction générale de l’alimentation” as part of the French Ministry of Agriculture, Food and Forestry. [file Data_Sheet_2.PDF]

For all studied outbreaks, the epidemiological links between cases and consumption of incriminated food products were established based on interviews of the French national public health agency (SpFrance).

Outbreak 1: This *S.* Typhimurium outbreak occurred in the department 23 (Creuse) potentially connected with the consumption during a family meal, on October 2011, of locally made pork products. Ten patients from 2 families were concerned. The set of strains selected includes 5 human strains isolated from department 23 (Creuse) and 3 strains from "meat products" (i.e. brawn, ham and rillettes) isolated from the family meal consumed. The main PFGE, MLVA and CRISPR profiles were STYMXB0215, 5-9-11-8-211 and 34, respectively.

Outbreak 2: This *S.* Typhimurium outbreak occurred in October 2014 in departments 19 (Corrèze) and 24 (Dordogne) connected with the consumption of eggs. The set of strains selected includes 9 human strains from the departments 19 and 24, 1 strain isolated from the meal consumed by patients in department 19 and 2 strains from environmental livestock producer of the offending eggs in department 24. The main MLVA and CRISPR profiles were 2-13-11-11-312 and 203, respectively.

Outbreak 3: This *S.* I 4,[5],12:i:- outbreak occurred between August and October 2011 in the departments 46 (Lot), 69 (Rhône) and 82 (Tarn-et-Garonne) connected with the consumption of pork products (meat and sausages). The set of strains selected for this outbreak includes 7 human strains isolated from the departments 46, 69 and 82, 5 strains isolated during September from pork products (mainly sausages) in a retail sale in department 46 and 9 strains isolated from pig carcasses (back, throat, outer thigh, chest and ham) from the slaughterhouse in department 82 from which the pork meat came. The main PFGE, MLVA and CRISPR profiles were STYMXB0126, 3-13-11--2-211 and 1, respectively.

Outbreak 4: Four *S.* I 4,[5],12:i:- outbreaks occurred between April and July 2011 through the departments 69 (Rhône), 73 (Savoie) and 74 (Haute-Savoie) connected with consumption of raw goat's milk cheese (these outbreaks were grouped in a unique epidemical event n°28/04/2011). This epidemical event concerned 12 patients whose one death (a person in later life). The set of strains selected includes 10 human strains isolated from departments 69, 73 and 74 (including the isolate from deceased patient) and 12 strains from raw milk cheese, milk and sheep goat from a farm in Haute Savoie (74). The main PFGE, MLVA and CRISPR profiles were STYMXB0126, 3-13-9--2-211 and 253, respectively.
