# Supplementary figures and images for: A Simple and Robust Statistical Method to Define Genetic Relatedness of Samples Related to Outbreaks at the Genomic Scale – Application to Retrospective Salmonella Foodborne Outbreak Investigations
Source: Front Microbiol. 2019 Oct 24;10:2413. doi: 10.3389/fmicb.2019.02413 (PMC6821717; doi:10.3389/fmicb.2019.02413)

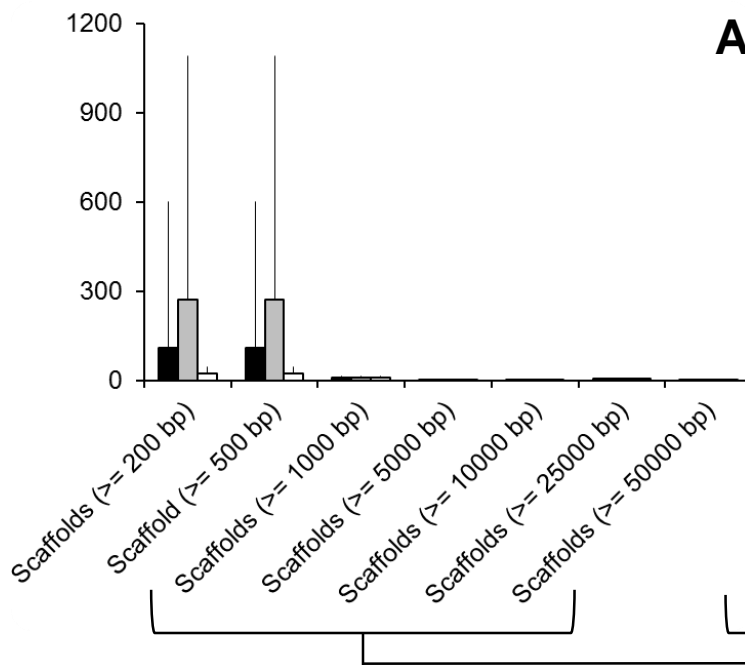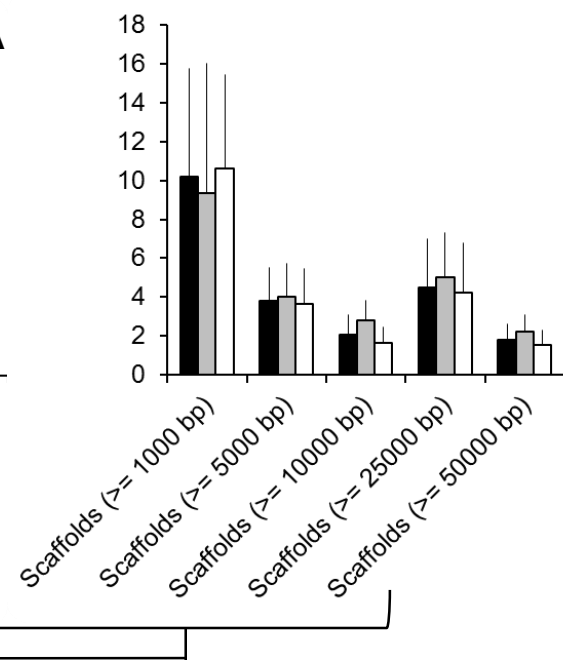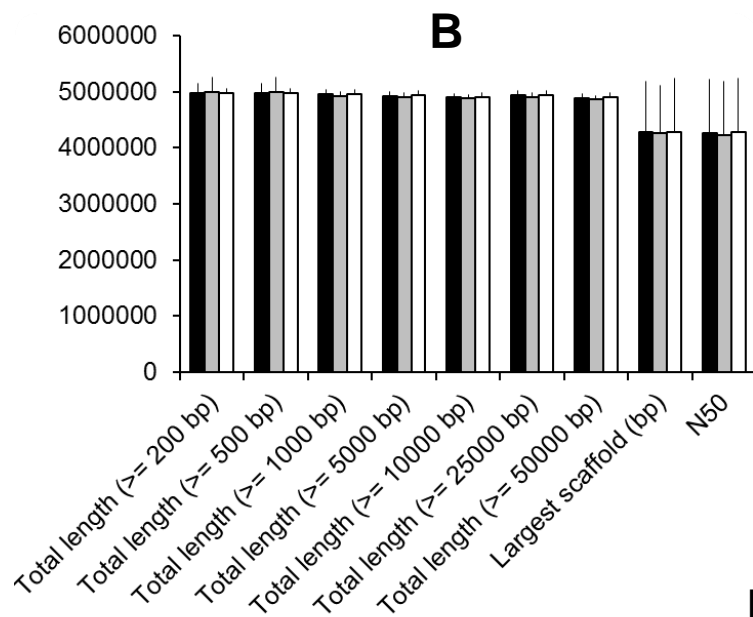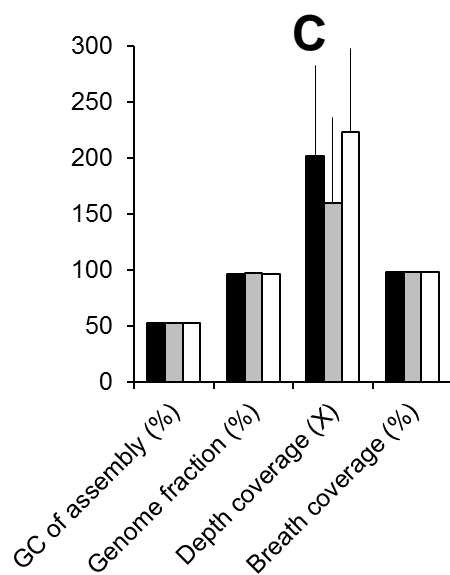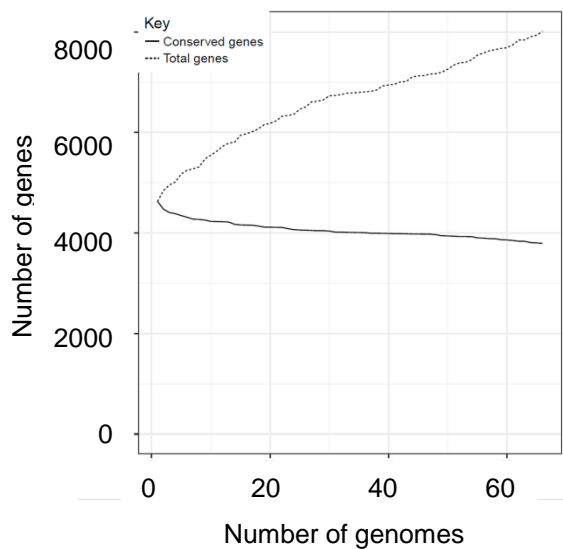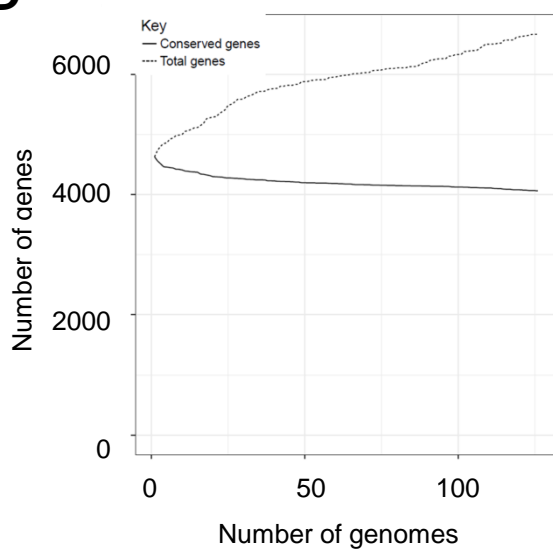

Supplement: DATA S3 — Number (A) and size (B) of scaffolds, other parameters of assembly and mapping (C) and number of genes resulting from pangenome analyses (D) of Salmonella enterica subsp. enterica (i.e., black bars; n = 192) serovars Typhimurium (i.e., gray bars or on the left side; n = 66) and S. 1,4,[5],12:i:- (i.e., white bars or on the right side; n = 126). Assembly, mapping and variant calling, as well as computing of quality metrics and pangenome analyses were performed with ARTWork, iVARCall2, Quast-MultiQC and Roary, respectively. Means and standard deviation are represented. [file Data_Sheet_3.PDF]
