# Supplementary material for: A Simple and Robust Statistical Method to Define Genetic Relatedness of Samples Related to Outbreaks at the Genomic Scale – Application to Retrospective Salmonella Foodborne Outbreak Investigations
Source: Front Microbiol. 2019 Oct 24;10:2413. doi: 10.3389/fmicb.2019.02413 (PMC6821717; doi:10.3389/fmicb.2019.02413)

Approach 'SNPs-1' (i.e. with recombination events)

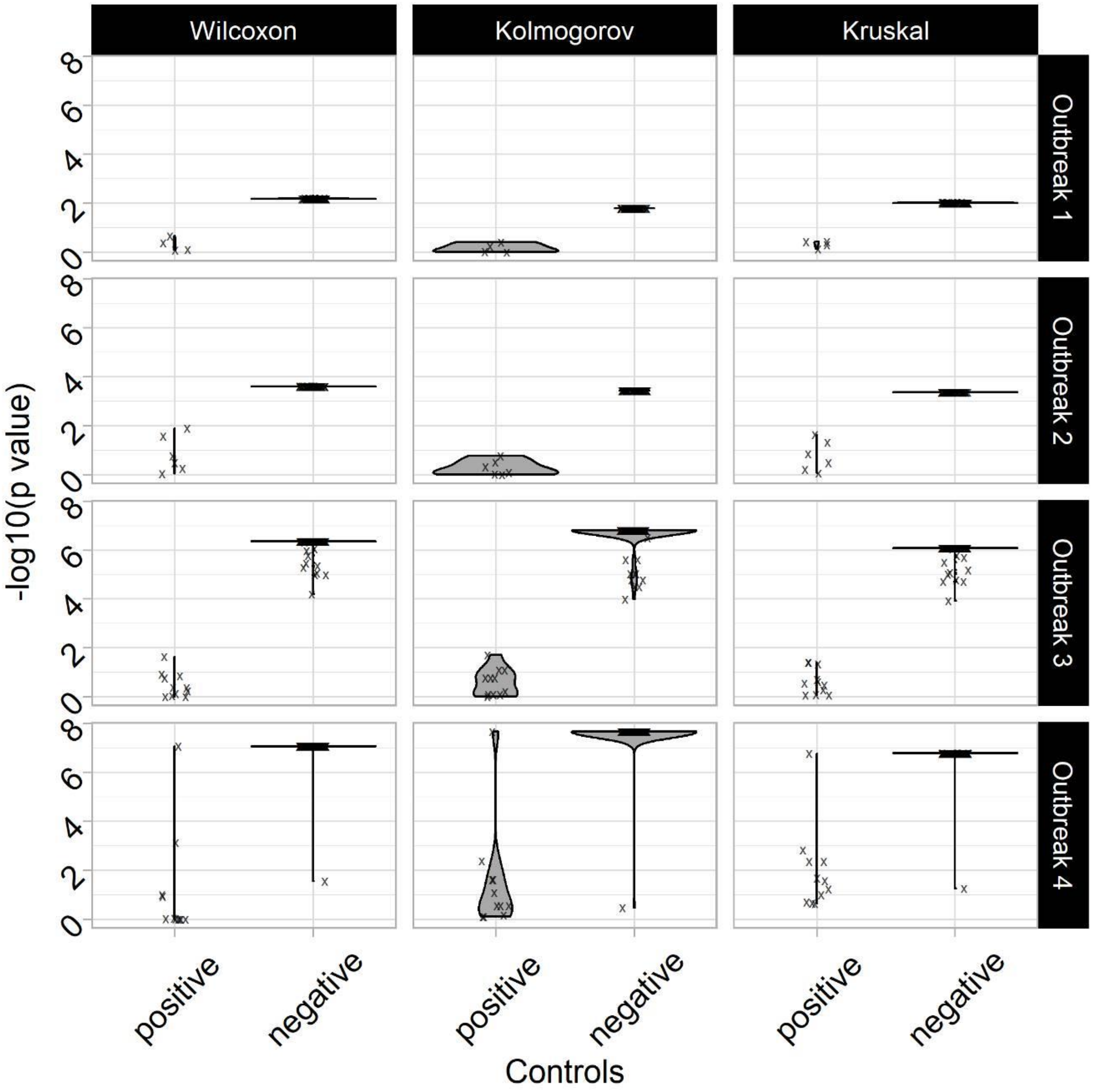

Approach 'SNPs-2' (i.e. without recombination events)

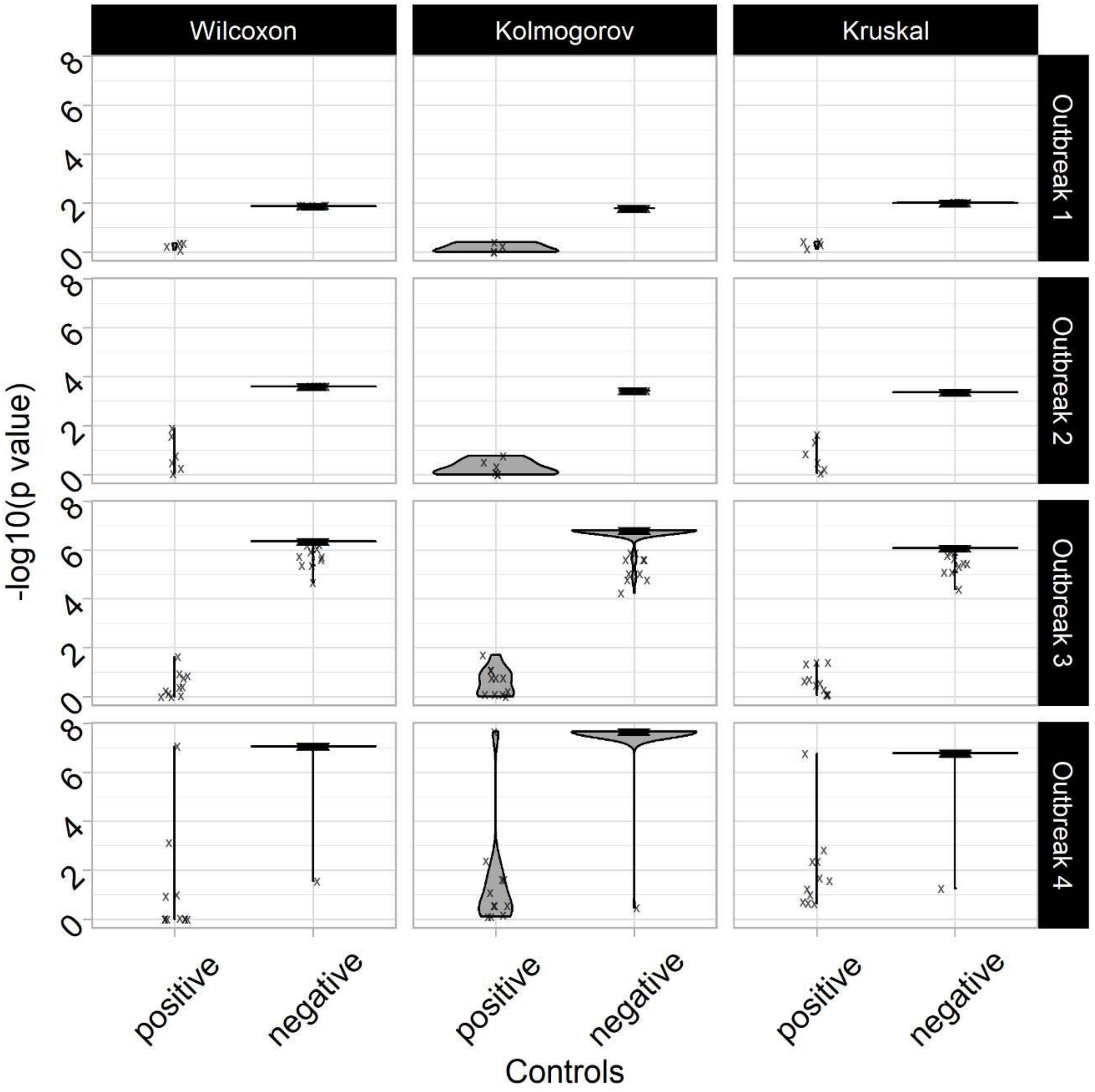

Approach ‘genes’

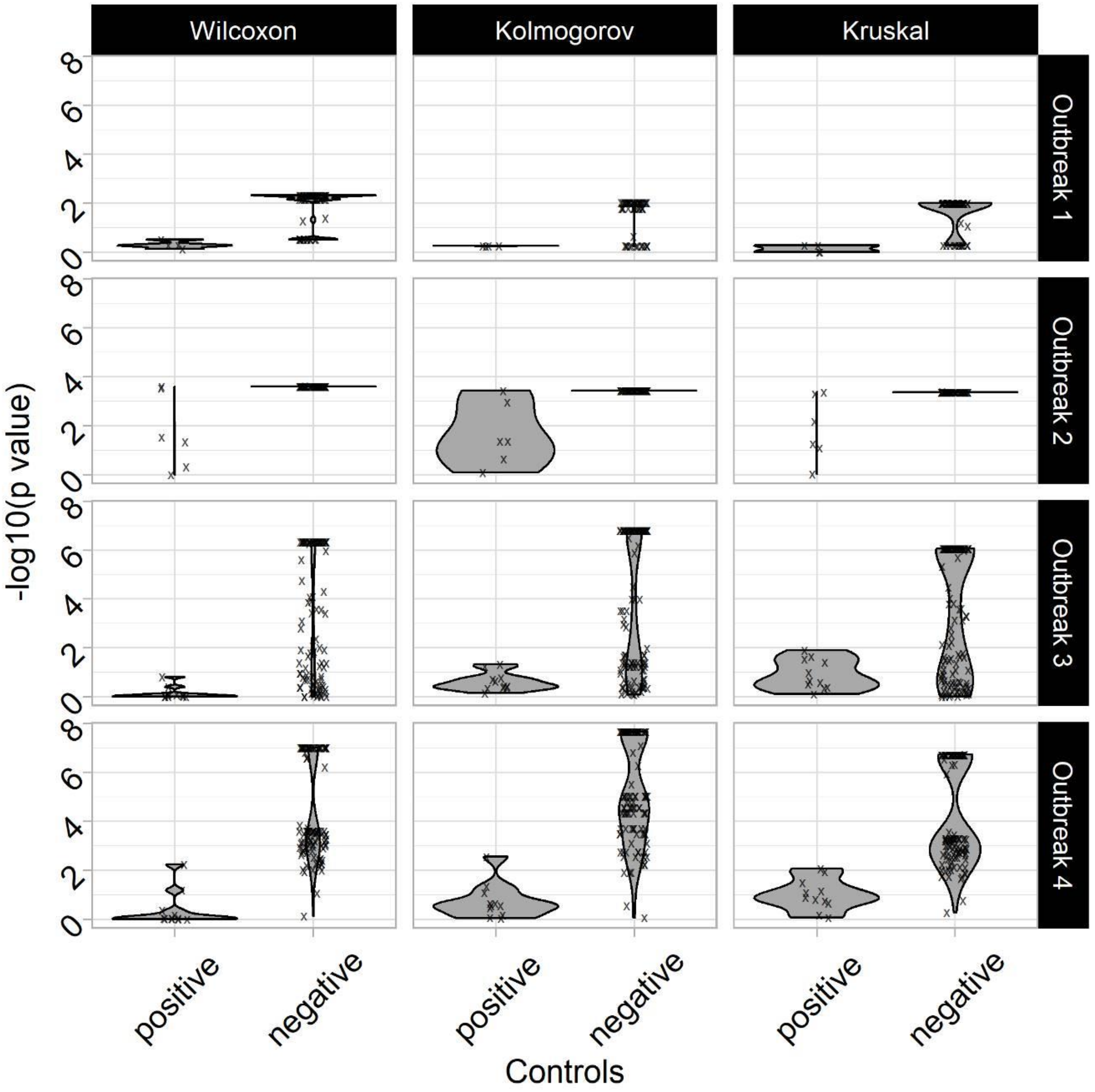

Approach 'kmers'

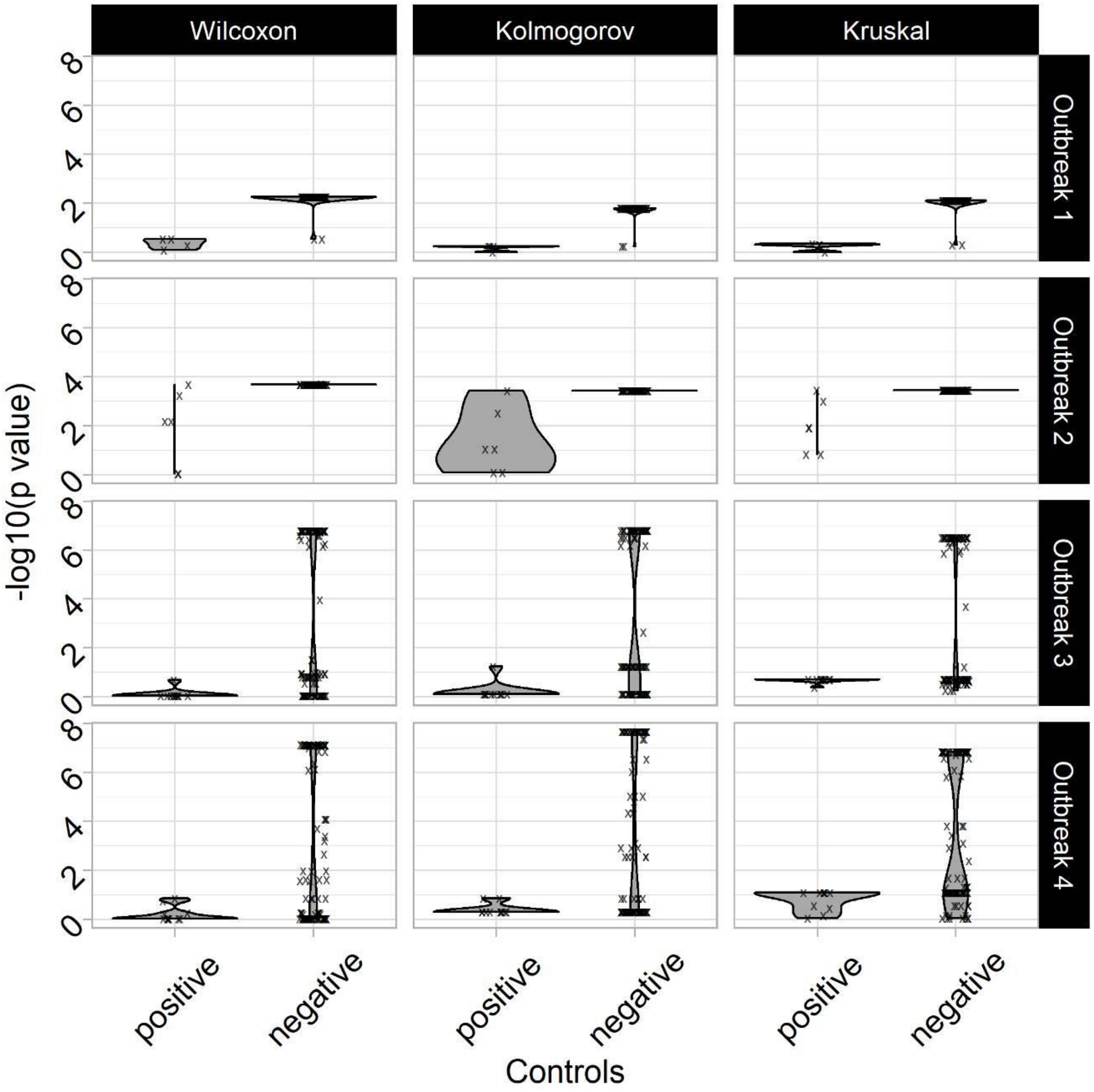

Approach ‘cgMLST’

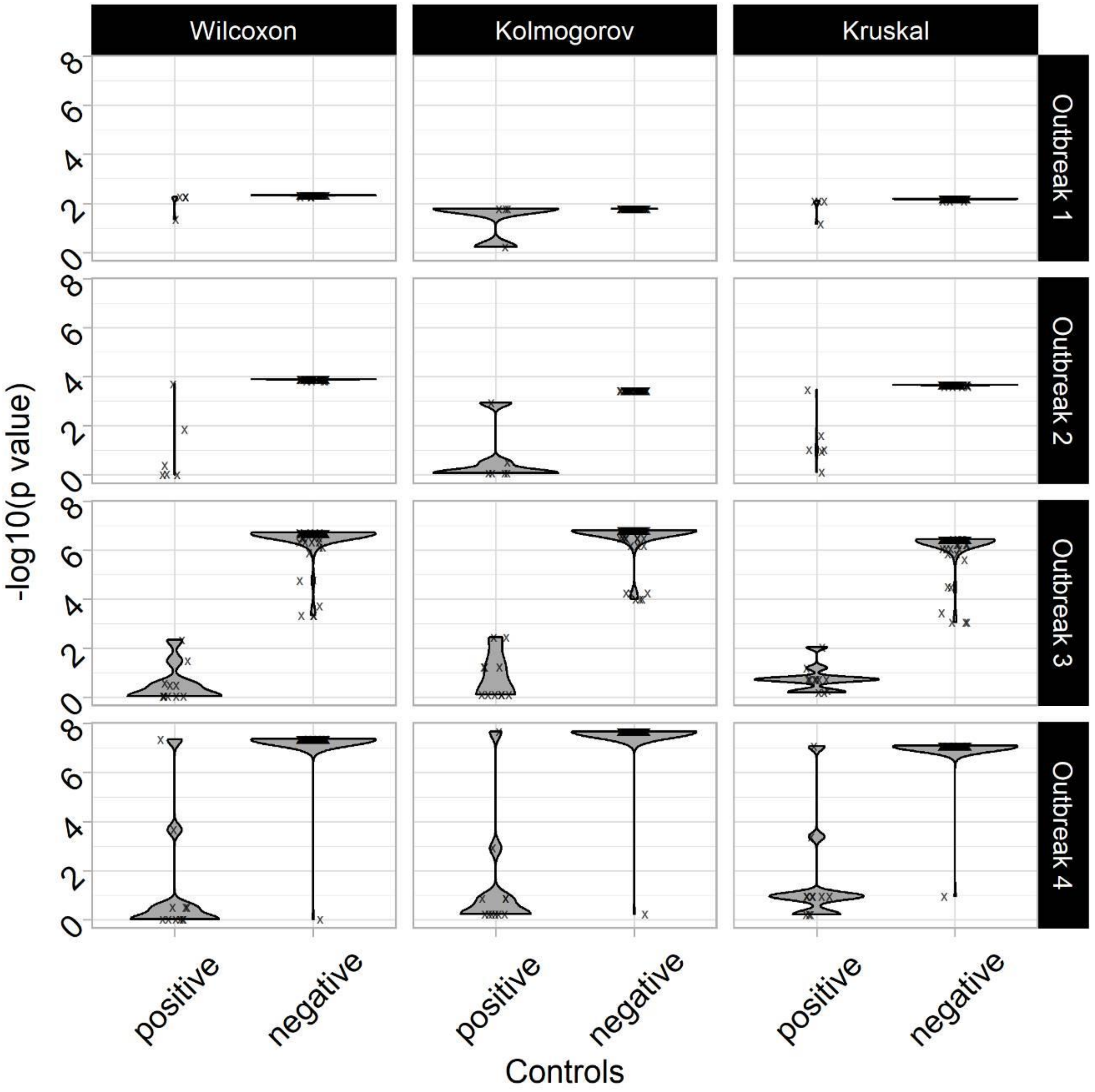

Approach ‘wgMLST’

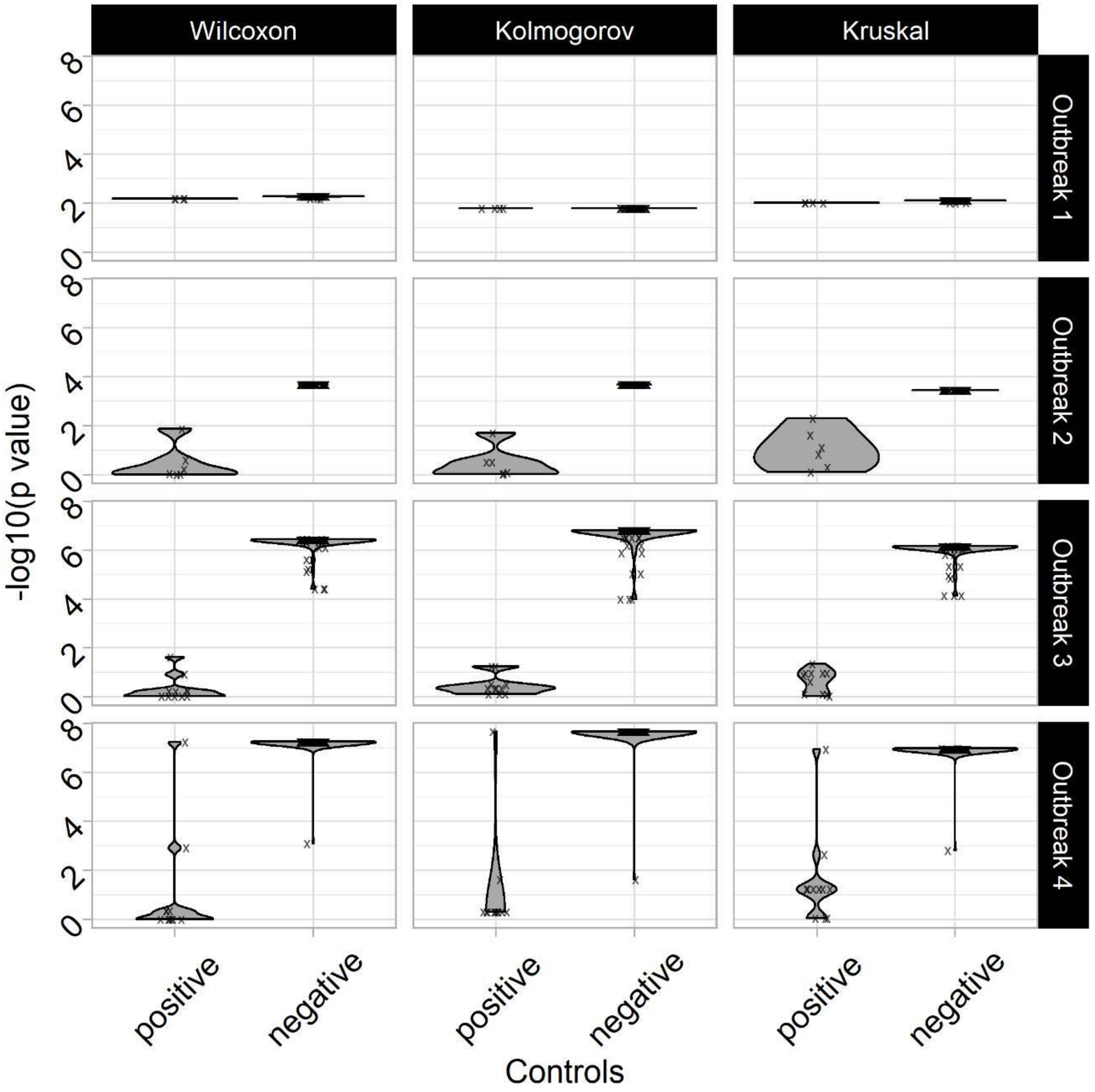

Supplement: DATA S4 — Likelihoods of non-parametric tests WS (i.e., differences of median values), KS (i.e., differences in distributions) and KW (i.e., differences of mean ranks) assessing statistical differences of pairwise differences of genomic features at the core (i.e., approaches ‘SNPs-1,’ ‘SNP-2,’ and ‘cgMLST’), accessory (i.e., approaches ‘genes’) and pangenome (i.e., approaches ‘kmers’ and ‘wgMLST’) scales in order to investigate food poisoning outbreaks of 192 S. Typhimurium (i.e., outbreaks #1 and #2; n = 66) and S. 1,4,[5],12:i:- (i.e., outbreaks #3 and #4; n = 126). The ‘SNPs’ approaches were performed including (i.e., ‘SNPs-1’) or excluding (i.e., ‘SNPs-2’) SNPs from recombination events identified with ClonalFrameML. The R script ‘matrix2association’ estimates statistical differences between two lists of pairwise differences existing across all genomes known to be involved in a studied outbreak (i.e., outbreak test set: TS) and between these genomes and a tested genome (i.e., outbreak control C+ or non-outbreak control C−) in order to assign (i.e., absence of statistical differences: H0 conserved), or not (i.e., presence of statistical differences: H0 rejected), this tested genome to the outbreak of interest. The approaches ‘SNPs-1,’ ‘SNP-2,’ ‘genes,’ ‘kmers,’ ‘cgMLST,’ and ‘wgMLST’ were performed with the workflows iVARCall2 with or without ClonalFrameML, ARTWork-Roary, ARTWork-QuickPhylo, and BioNumerics (Applied Maths), respectively. [file Data_Sheet_4.PDF]
