# Supplementary material for: A Simple and Robust Statistical Method to Define Genetic Relatedness of Samples Related to Outbreaks at the Genomic Scale – Application to Retrospective Salmonella Foodborne Outbreak Investigations
Source: Front Microbiol. 2019 Oct 24;10:2413. doi: 10.3389/fmicb.2019.02413 (PMC6821717; doi:10.3389/fmicb.2019.02413)

Samples randomly selected and included in the outbreak test set (i.e. TS)

Outbreak #1			
**Trial 1**	**Trial 2**	**Trial 3**	**Trial 4**
11CEB5897SAL	11CEB5898SAL	2011_10159	2011_10163
11CEB5898SAL	2011_10159	11CEB5898SAL	2011_10162
11CEB5899SAL	2011_10162	2011_10162	2011_10160
2011_10160	11CEB5899SAL	2011_10163	2011_10159
**Outbreak #2**			
**Trial 1**	**Trial 2**	**Trial 3**	**Trial 4**
2014LSAL04863	2014LSAL04593	2014_10613	2014_10611
2014LSAL05090	2014_10615	2014_10575	2014_10516
2014_10610	2014LSAL05090	2014_10612	2014_10613
2014_10611	2014LSAL04863	2014_10610	2014LSAL05090
2014_10614	2014_10610	2014LSAL04863	2014_10575
2014_10615	2014_10613	2014LSAL05090	2014LSAL04593
**Outbreak #3**			
**Trial 1**	**Trial 2**	**Trial 3**	**Trial 4**
11CEB4793SAL	11CEB6571SAL	11CEB4113SAL	11CEB6568SAL
11CEB6549SAL	11CEB6568SAL	2011_06755	2011_09295
11CEB6555SAL	11CEB4791SAL	11CEB6568SAL	11CEB6561SAL
11CEB6557SAL	11CEB6549SAL	11CEB4791SAL	11CEB4686SAL
11CEB6561SAL	2011_06602	2011_08649	11CEB4791SAL
11CEB6568SAL	2011_08648	11CEB6575SAL	2011_08649
11CEB6571SAL	11CEB6561SAL	2011_08648	11CEB6557SAL
2011_06755	11CEB6575SAL	2011_08647	2011_06755
2011_08647	11CEB6557SAL	11CEB6573SAL	11CEB6555SAL
2011_09295	2011_08647	11CEB4686SAL	2011_08647
**Outbreak #4**			
**Trial 1**	**Trial 2**	**Trial 3**	**Trial 4**
11CEB2164SAL	11CEB2164SAL	11CEB2911SAL	11CEB2911SAL
11CEB2250SAL	11CEB2326SAL	2011_01997	2011_01883
11CEB2251SAL	2011_01883	11CEB3431SAL	2011_02231
11CEB2326SAL	2011_01989	2011_02231	2011_01989
11CEB2816SAL	11CEB2911SAL	11CEB2164SAL	2011_02192
11CEB3431SAL	2011_02228	11CEB3015SAL	11CEB3431SAL
2011_02192	2011_01997	2011_01883	11CEB3440SAL
2011_02227	11CEB2250SAL	2011_01986	11CEB2164SAL
2011_02228	2011_02155	11CEB2816SAL	2011_01986
2011_02230	11CEB2817SAL	2011_02228	2011_02228
2011_02231	11CEB3440SAL	11CEB3440SAL	11CEB2817SAL

Approach ‘SNPs-1’ (i.e. with recombination events) applied on outbreak #1

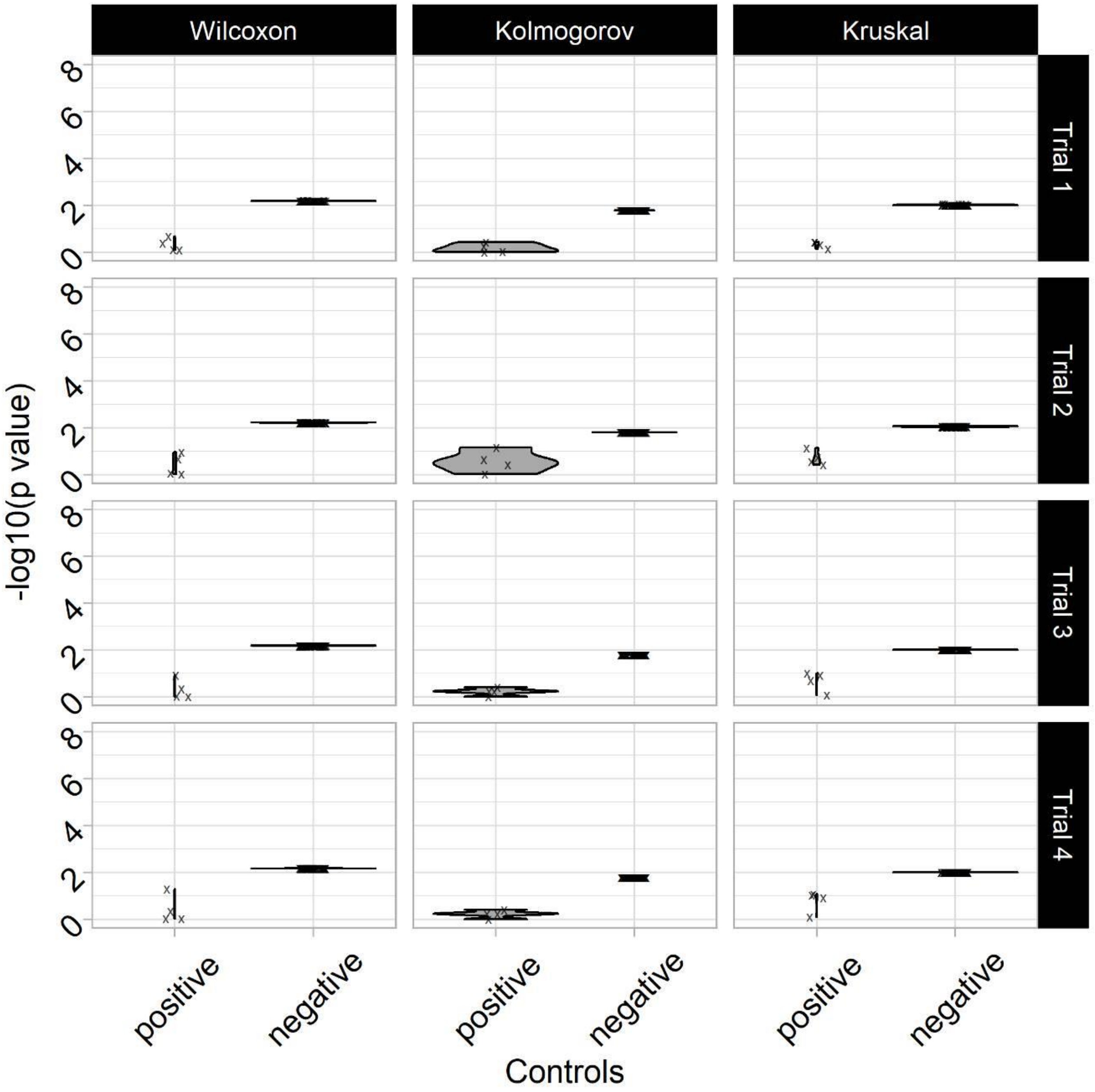

Approach 'SNPs-1' (i.e. with recombination events) applied on outbreak #2

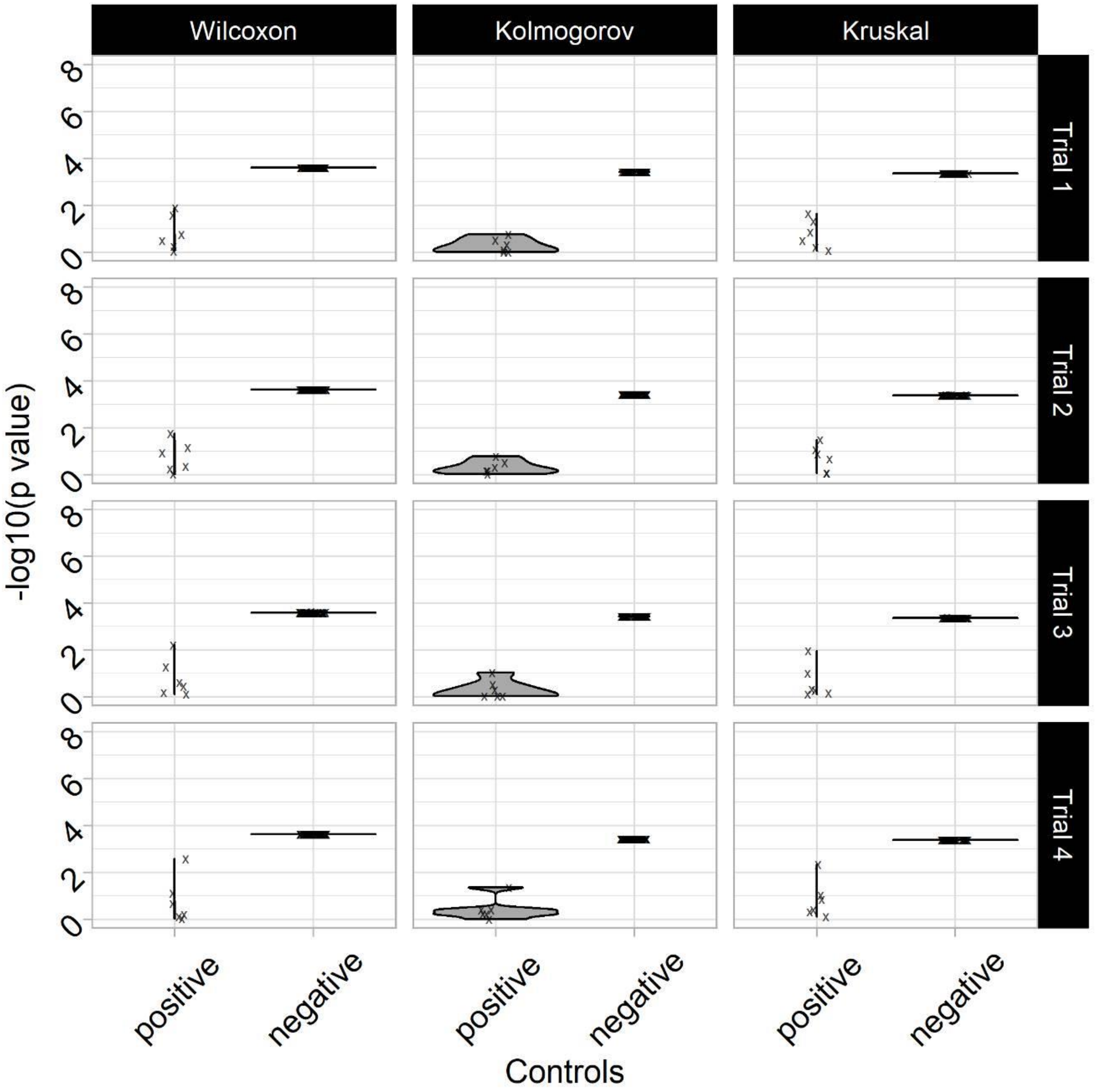

Approach 'SNPs-1' (i.e. with recombination events) applied on outbreak #3

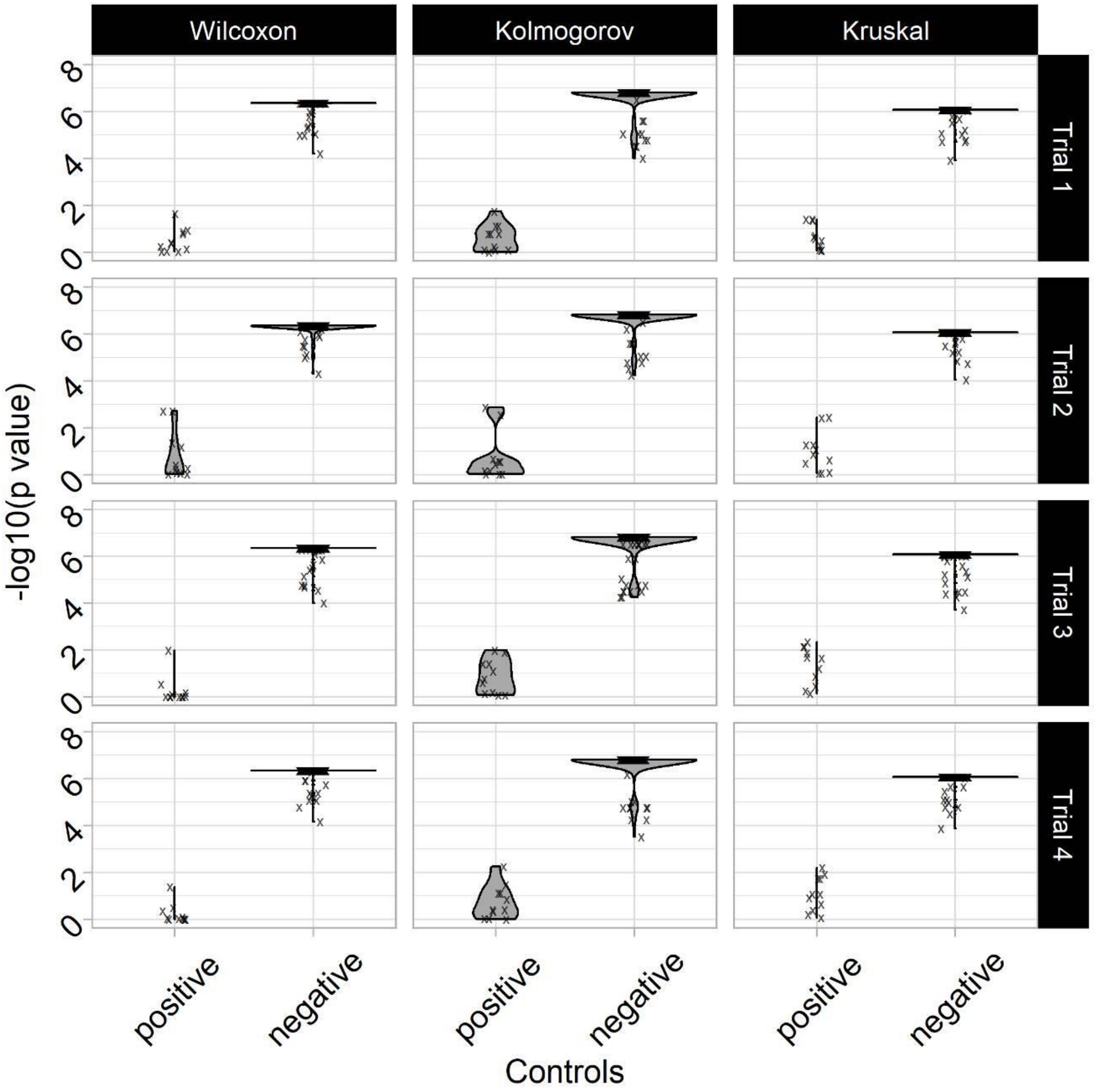

Approach 'SNPs-1' (i.e. with recombination events) applied on outbreak #4

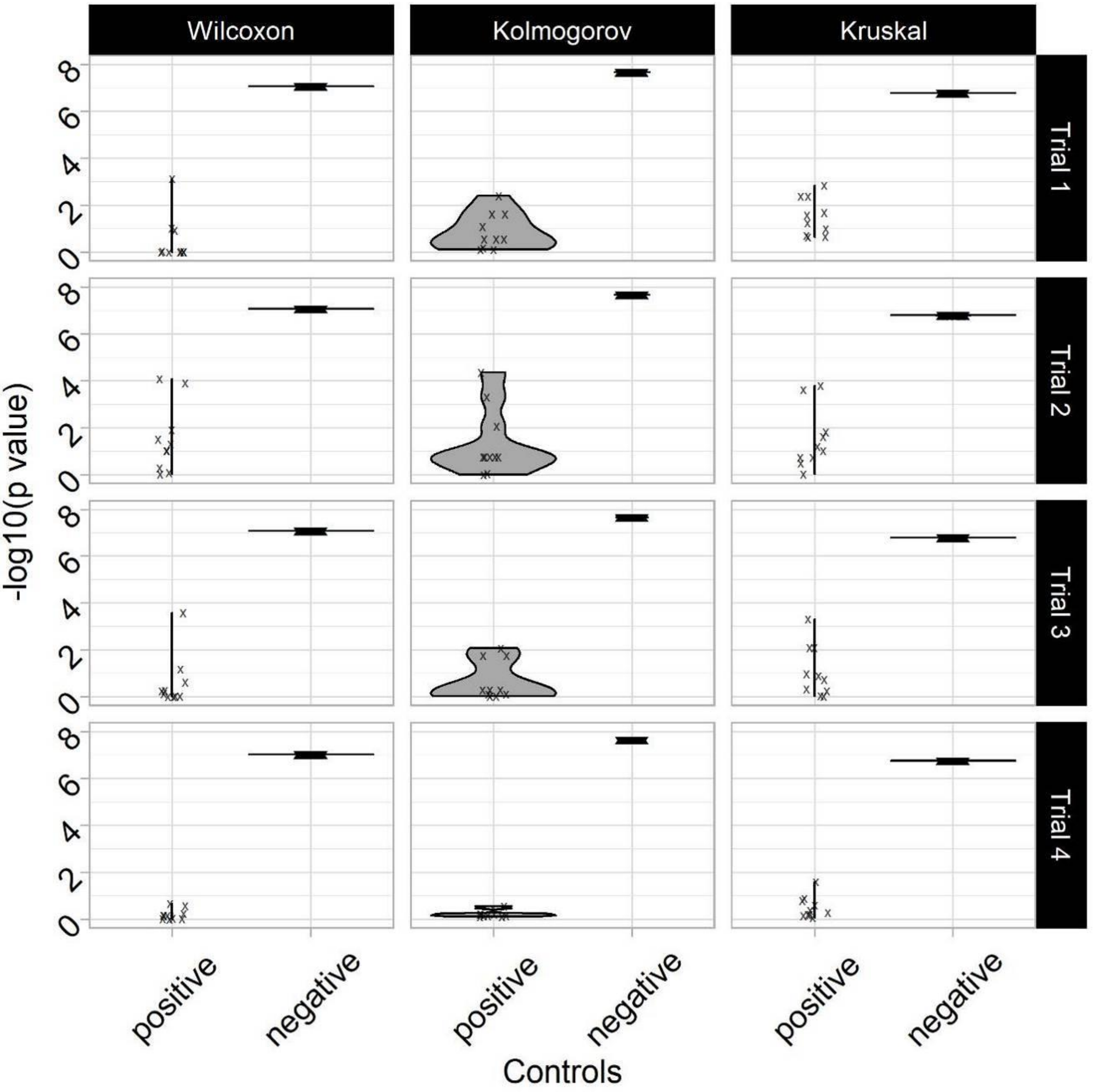

Supplement: DATA S5 — Reproducibility of negative common logarithms of p-values from non-parametric tests WS (i.e., differences of median values), KS (i.e., differences in distributions) and KW (i.e., differences of mean ranks) assessing the statistical differences of pairwise SNP differences including recombination events (i.e., approach ‘SNP-1’) in order to investigate food poisoning outbreaks of 192 S. Typhimurium (i.e., outbreaks #1 and #2; n = 66) and S. 1,4,[5],12:i:- (i.e., outbreaks #3 and #4; n = 126). The R script ‘matrix2association’ estimates statistical differences between two lists of pairwise differences existing across all genomes known to be involved in a studied outbreak (i.e., outbreak test set: TS) and between these genomes and a tested genome (i.e., outbreak control C+ or non-outbreak control C−) in order to assign (i.e., absence of statistical differences: H0 conserved), or not (i.e., presence of statistical differences: H0 rejected), this tested genome to the outbreak of interest. The approach ‘SNPs-1’ was performed with the workflow iVARCall2. In total, four random selections of samples included in the outbreak test set (TS) were performed in order to access reproducibility of the non-parametric approaches. [file Data_Sheet_5.PDF]
