# Supplementary material for: A Simple and Robust Statistical Method to Define Genetic Relatedness of Samples Related to Outbreaks at the Genomic Scale – Application to Retrospective Salmonella Foodborne Outbreak Investigations
Source: Front Microbiol. 2019 Oct 24;10:2413. doi: 10.3389/fmicb.2019.02413 (PMC6821717; doi:10.3389/fmicb.2019.02413)
Supplement: TABLE S4 — Mean, standard deviation (i.e., the signs ‘±’), minimum and maximum (i.e., in square brackets) of the negative common logarithms of p-values from non-parametric tests WS (i.e., differences of median values), KS (i.e., differences in distributions), and KW (i.e., differences of mean ranks) assessing statistical differences of pairwise differences of genomic features at the core (i.e., approaches ‘SNPs-1,’ ‘SNPs-2,’ and ‘cgMLST’), accessory (i.e., approaches ‘genes’) and pangenome (i.e., approaches ‘kmers’ and ‘wgMLST’) scales in order to investigate food poisoning outbreaks of 192 S. Typhimurium (i.e., outbreaks #1 and #2; n = 66) and S. 1,4,[5],12:i:- (i.e., outbreaks #3 and #4; n = 126). The ‘SNP’ approaches were performed including (i.e., ‘SNPs-1’) or excluding (i.e., ‘SNPs-2’) SNPs from recombination events identified with ClonalFrameML. The R script ‘matrix2association’ estimates statistical differences between two lists of pairwise differences existing across all genomes known to be involved in a studied outbreak (i.e., outbreak tested set: TS) and between these genomes and a tested genome (i.e., outbreak control C+ or non-outbreak control C−) in order to assign (i.e., absence of statistical differences: H0 conserved), or not (i.e., presence of statistical differences: H0 rejected), this tested genome to the outbreak of interest. The approaches ‘SNPs-1,’ ‘SNPs-2,’ ‘genes,’ ‘kmers,’ ‘cgMLST,’ and ‘wgMLST’ were performed with the workflows iVARCall2 with and without ClonalFrameML, ARTWork-Roary, ARTWork-QuickPhylo, and BioNumerics (Applied Maths), respectively. [file Table_4.PDF]

Outbreak*	Test	Positive control (C+) and negative control (C-)	Approach					
			SNPs-1	SNPs-2	genes	kmers	cgMLST	wgMLST
# 1	Wilcoxon rank sum	C+	0.31±0.21 [0.00,0.41]	0.26±0.10 [0.08,0.36]	0.29±0.11 [0.13,0.42]	0.36±0.18 [0.09,0.54]	2.04±0.34 [1.36,2.26]	2.18±0.00 [2.18,2.19]
		C-	2.18±0.01 [2.17,2.20]	1.88±0.00 [1.87,1.90]	1.88±0.60 [0.52,2.32]	2.20±0.11 [0.54,2.26]	2.33±0.01 [2.26,2.33]	2.27±0.01 [2.18,2.27]
	Kolmogorov-Smirnov	C+	0.17±0.16 [0.08,0.66]	0.17±0.16 [0.00,0.41]	0.25±0.01 [0.23,0.26]	0.17±0.09 [0.09,0.23]	1.40±0.58 [0.23,1.78]	1.78±0.00 [1.78,1.78]
		C-	1.78±0.00 [1.78,1.78]	1.78±0.00 [1.78,1.78]	1.55±0.61 [0.23,2.02]	1.73±0.10 [0.23,1.78]	1.78±0.00 [1.78,1.78]	1.78±0.00 [1.78,1.78]
	Kruskal-Wallis	C+	0.32±0.10 [0.13,0.42]	2.01±0.00 [2.00,2.04]	0.14±0.14 [0.00,0.28]	0.23±0.12 [0.00,0.34]	1.87±0.35 [1.17,2.10]	2.01±0.01 [2.01,2.02]
		C-	2.01±0.01 [2.00,2.04]	0.32±0.10 [0.13,0.42]	1.60±0.58 [0.28,1.99]	2.04±0.12 [0.30,2.10]	2.17±0.01 [2.10,2.17]	2.11±0.01 [2.01,2.11]
# 2	Wilcoxon rank sum	C+	0.83±0.60 [0.03,1.89]	0.83±0.60 [0.03,1.89]	0.71±0.23 [0.00,3.59]	1.88±1.23 [0.03,3.68]	1.00±1.19 [0.02,3.71]	0.45±0.52 [0.00,1.87]
		C-	3.60±0.00 [3.59,3.60]	3.60±0.00 [3.59,3.60]	3.59±0.00 [3.59,3.61]	3.68±0.00 [3.68,3.69]	3.89±0.01 [3.83,3.89]	3.68±0.00 [3.68,3.68]
	Kolmogorov-Smirnov	C+	0.28±0.25 [0.00,0.76]	0.28±0.25 [0.00,0.76]	1.63±1.03 [0.08,3.42]	1.36±1.07 [0.08,3.42]	0.63±0.77 [0.08,2.94]	0.47±0.43 [0.01,1.70]
		C-	3.42±0.00 [3.42,3.42]	3.42±0.00 [3.42,3.42]	3.42±0.00 [3.42,3.42]	3.42±0.00 [3.42,3.42]	3.42±0.00 [3.42,3.42]	3.68±0.00 [3.68,3.68]
	Kruskal-Wallis	C+	0.75±0.51 [0.06,1.64]	0.75±0.51 [0.06,1.64]	1.86±1.08 [0.01,3.35]	1.98±0.82 [0.82,3.44]	1.37±0.78 [0.11,3.48]	1.04±0.63 [0.11,2.28]
		C-	3.36±0.00 [3.35,3.36]	3.36±0.00 [3.35,3.36]	3.35±0.00 [3.35,3.37]	3.44±0.00 [3.44,3.46]	3.66±0.01 [3.59,3.66]	3.45±0.00 [3.45,3.45]
# 3	Wilcoxon rank sum	C+	0.49±0.41 [0.01,1.65]	0.50±0.41 [0.01,1.65]	0.14±0.17 [0.00,0.81]	0.10±0.11 [0.04,0.68]	0.51±0.52 [0.04,2.33]	0.33±0.35 [0.02,1.63]
		C-	6.25±0.18 [4.20,6.35]	6.29±0.12? [4.67,6.35]	3.39±2.48 [0.00,6.33]	3.20±3.09 [0.04,6.77]	6.49±0.33 [3.35,6.71]	6.31±0.19 [4.42,6.44]
	Kolmogorov-Smirnov	C+	0.61±0.47 [0.00,1.72]	0.61±0.47 [0.00,1.72]	0.62±0.29 [0.15,1.32]	0.22±0.19 [0.12,1.25]	0.85±0.80 [0.12,2.45]	0.47±0.29 [0.12,1.25]
		C-	6.62±0.33 [4.00,6.81]	6.65±0.27 [4.25,6.81]	3.57±2.66 [0.09,6.81]	3.30±3.00 [0.12,6.81]	6.61±0.33 [4.00,6.81]	6.64±0.28 [4.00,6.81]
	Kruskal-Wallis	C+	0.64±0.42 [0.07,1.41]	0.65±0.42 [0.07,1.41]	0.92±0.53 [0.11,1.91]	0.68±0.05 [0.39,0.71]	0.75±0.32 [0.20,2.05]	0.64±0.41 [0.02,1.34]
		C-	5.97±0.18 [3.92,6.07]	6.01±0.12 [4.39,6.07]	3.23±2.37 [0.00,6.05]	3.22±2.82 [0.26,6.49]	6.21±0.34 [3.06,6.43]	6.03±0.19 [4.14,6.17]
# 4	Wilcoxon rank sum	C+	0.52±0.70 [0.00,3.13]	0.52±0.70 [0.00,3.13]	0.19±0.23 [0.00,1.19]	0.22±0.24 [0.02,0.85]	0.54±0.63 [0.02,3.67]	0.40±0.51 [0.01,2.93]
		C-	7.06±0.00 [7.06,7.07]	7.06±0.00 [7.06,7.07]	4.36±1.81 [0.13,7.00]	2.94±3.01 [0.02,7.13]	7.34±0.00 [7.34,7.34]	7.23±0.00 [7.23,7.26]
	Kolmogorov-Smirnov	C+	0.88±0.64 [0.11,2.39]	0.88±0.64 [0.11,2.39]	0.56±0.30 [0.04,1.34]	0.41±0.18 [0.30,0.86]	0.69±0.55 [0.23,2.92]	0.43±0.24 [0.30,1.62]
		C-	7.66±0.00 [7.66,7.66]	7.66±0.00 [7.66,7.66]	5.06±1.72 [0.07,7.66]	3.29±3.10 [0.30,7.66]	7.66±0.00 [7.66,7.66]	7.66±0.00 [7.66,7.66]
	Kruskal-Wallis	C+	1.51±0.66 [0.64,2.84]	1.51±0.66 [0.64,2.84]	0.91±0.43 [0.06,2.07]	0.77±0.38 [0.03,1.08]	0.98±0.48 [0.22,3.39]	1.01±0.59 [0.03,2.64]
		C-	6.78±0.00 [6.78,6.78]	6.78±0.00 [6.78,6.78]	4.08±1.81 [0.27,6.72]	3.15±2.62 [0.03,6.85]	7.06±0.00 [7.06,7.09]	6.95±0.00 [6.95,6.98]

* n = Outbreak #1, #2, #3 and #4 include 4, 6, 10 and 11 confirmed cases; 4, 6, 11 and 11 outbreak positive controls; as well as 58, 54, 105 and 104 non-outbreak negative controls, respectively. The samples 2013LSAL03045 (i.e. expected C- and identified as C+) and 11CEB5591SAL (i.e. expected C+ and identified as C-) from the outbreak #4 were not included in calculation.
